# The UBE2/E2 ubiquitin-conjugating enzyme family at the interface of tumor biology and antitumor immunity: mechanisms, biomarkers, and therapeutic opportunities

**DOI:** 10.3389/fimmu.2026.1847760

**Published:** 2026-06-10

**Authors:** Hai Zhao, Jiaxin Yang, Fan Yang

**Affiliations:** 1 Surgical Skills Training Laboratory, Clinical Practice Teaching Center, Shenyang Medical College, Shenyang, China; 2 Department of Anesthesiology, Shengjing Hospital of China Medical University, Shenyang, China

**Keywords:** biomarker, cancer immunopharmacology, immune evasion, tumor immunity, UBE2 enzymes, ubiquitination

## Abstract

While the catalytic role of ubiquitin-conjugating (UBE2/E2) enzymes within the ubiquitin–proteasome system is well established, their substrate-specifying functions in cancer, particularly at the tumor–immune interface, remain comparatively underexplored. In this review, we provide an integrative re-evaluation of the UBE2 family organized around three concepts that, we argue, distinguish recent UBE2 research from earlier descriptive accounts: (i) tumor-context–dependent functional duality, in which the same UBE2 enzyme exerts opposing effects across tissue types and substrate landscapes; (ii) convergence of mechanistically distinct UBE2 members on a small set of shared signaling and immunometabolic nodes; and (iii) the emerging role of UBE2 enzymes at the tumor–immune interface, directly linking UBE2 biology to contemporary immunotherapy paradigms. Building on this framework, we synthesize recent evidence showing how dysregulated UBE2 members shape major cancer hallmarks, including uncontrolled proliferation, oncogenic signaling, metabolic reprogramming, immune evasion, and therapeutic resistance, while emphasizing that most reported phenotypes are derived from a limited set of tumor lineages and should not be uncritically extrapolated to all malignancies. Across tumor settings, multiple UBE2 members converge on PI3K/AKT, MAPK/ERK, NF-κB, HIF-1α, antigen-presentation pathways, checkpoint-ligand regulation, and macrophage polarization. We further discuss the potential of UBE2-based signatures as candidate prognostic and immune-stratification biomarkers. While these associations are biologically compelling, most derive from retrospective cohorts and require prospective, multi-center validation before clinical consideration. We also evaluate current pharmacological strategies targeting UBE2-driven vulnerabilities, including small-molecule inhibitors, natural compounds, and combination approaches with chemotherapy and immune checkpoint blockade. Collectively, this synthesis positions UBE2 enzymes as context-resolved regulators of tumor progression and the immune microenvironment, with growing potential for biomarker-guided therapeutic exploitation in precision cancer immunopharmacology, subject to rigorous prospective validation.

## 1 Introduction

Cancer remains a major global cause of morbidity and mortality, with approximately 20 million new cases and 9.7 million deaths estimated worldwide in 2022 (1). Although kinase-targeted therapies and immune-checkpoint inhibitors have transformed treatment paradigms in multiple malignancies, durable disease control is often undermined by tumor adaptation and signaling plasticity (2, 3). These limitations have intensified interest in alternative regulatory layers beyond canonical kinase cascades, among which ubiquitination has emerged as a highly versatile and potentially druggable determinant of oncogenic cell fate.

The ubiquitin–proteasome system (UPS) is a tightly regulated and evolutionarily conserved machinery that governs selective protein turnover and broader protein-quality control in eukaryotic cells (4, 5). Through sequential enzymatic reactions involving ubiquitin-activating (E1), ubiquitin-conjugating (E2), and ubiquitin ligase (E3) enzymes, ubiquitin is attached to target proteins to regulate their stability, localization, activity, and interactions (6, 7). Because ubiquitination is one of the most abundant post-translational modifications in eukaryotic cells, the UPS influences a broad range of cellular processes, including cell-cycle progression, DNA damage responses, signal transduction, immune regulation, and adaptation to oxidative stress (7–16). Within this system, the ubiquitin-conjugating enzymes encoded by the UBE2 family occupy a central position in ubiquitin transfer. The human UBE2 superfamily comprises approximately 35–40 members and has increasingly been recognized as more than a passive intermediary between E1 and E3 enzymes (17–20). Instead, accumulating evidence indicates that UBE2 enzymes can actively shape the specificity, efficiency, and signaling consequences of ubiquitination, thereby influencing diverse biological outputs.

In cancer, dysregulation of UBE2 enzymes has emerged as a recurrent and multifaceted feature of tumor progression (21, 22). Aberrant expression or activity of specific UBE2 members has been linked to sustained proliferative signaling (23, 24), evasion of apoptosis (25), metabolic reprogramming (26), and metastatic dissemination (27). In parallel, a growing body of evidence suggests that UBE2-dependent ubiquitin signaling may also intersect with the tumor–immune interface by influencing antigen processing, inflammatory signaling, and immune-cell recruitment (22, 28). However, compared with their better-characterized tumor-intrinsic functions, the contribution of UBE2 enzymes to tumor–immune crosstalk remains less clearly defined and is often supported by context-dependent or correlative observations.

Although several valuable reviews have addressed individual UBE2 enzymes or specific cancer types (7, 17, 19), an integrated framework linking UBE2 substrate selection, chain-type specificity, and tumor–immune crosstalk is still lacking. The present review therefore departs from descriptive enumeration and is organized around three concepts that, in our view, capture what is genuinely novel in recent UBE2 oncology research. First, we apply a functional-duality lens, documenting how the same UBE2 enzyme can switch between oncogenic and tumor-suppressive output depending on tissue lineage, substrate availability, and E2–E3 partnership, challenging the single-enzyme/single-function narratives often adopted in earlier syntheses. Second, we map convergence points at which mechanistically distinct UBE2 members regulate a shared and relatively limited set of signaling and immunometabolic nodes, providing a pathway-level rather than enzyme-level rationale for intervention. Third, we explicitly position UBE2 enzymes at the tumor–immune interface, an axis underrepresented in previous UBE2-focused reviews but increasingly central to combination immunotherapy. By integrating mechanistic and translational evidence around these three axes, this review aims to move the field beyond a broad recognition of UBE2 enzymes as ‘active’ players, toward a context-resolved framework for potential biomarker-guided therapeutic exploitation in precision cancer immunopharmacology. It is important to note, however, that most biomarker associations reported to date derive from retrospective analyses and remain hypothesis-generating pending prospective validation.

## 2 Structure–function overview of the UBE2 family

The UBE2 family comprises the central catalytic relay of the ubiquitination cascade. Although E2 enzymes were historically viewed primarily as ubiquitin carriers shuttling activated ubiquitin from E1 enzymes to E3 ligases, structural and biochemical studies over the past two decades have refined this view by demonstrating that E2 enzymes contribute, in cooperation with their cognate E3 partners and substrate context, to substrate engagement, catalytic processivity, and ubiquitin chain-type selection (16, 29, 30). The extent to which these intrinsic regulatory contributions translate into pathophysiologically meaningful effects in cancer is, however, family-member– and context-dependent, a caveat that we revisit when interpreting the functional studies discussed below.

Human cells encode approximately 35 ubiquitin E2 enzymes, all of which share a conserved ubiquitin-conjugating (UBC) core domain of roughly 150 amino acids, while differing in N- and/or C-terminal extensions that contribute to localization, interaction selectivity, and regulation of catalytic output (16). Historically, these enzymes were classified into four groups according to the presence of such terminal extensions, with class I enzymes containing only the UBC core and class IV members, including UBE2O and BIRC6, representing markedly larger multidomain proteins (31). Structurally, the canonical UBC fold consists of an N-terminal α-helix, a four-stranded β-meander, a short 3_10-helix, a central cross-over helix, and two C-terminal α-helices (29, 30). The active-site cysteine lies within a shallow catalytic cleft and is typically supported by a conserved His-Pro-Asn motif, although selected family members display motif variation without complete loss of conjugating activity (32–34). Additional residues within the catalytic cleft, including a negatively charged residue in the α3-α4 loop, further contribute to positioning the incoming substrate lysine for catalysis (35–39).

Functionally, UBE2 enzymes accept activated ubiquitin from E1 enzymes, predominantly UBA1 and, in selected settings, UBA6, to generate the thioester-linked E2~Ub intermediate (40–42). Structural analyses of E2~Ub mimics have shown that this intermediate is conformationally dynamic and can adopt a compact “closed” state in which the Ile44 hydrophobic patch of ubiquitin engages conserved residues on the E2 cross-over helix (43–47). This conformational property is mechanistically important because it influences ubiquitin discharge and catalytic efficiency. In parallel, several UBE2 enzymes contribute directly to ubiquitin-chain topology. UBE2K promotes Lys48-linked chain formation, UBE2S directs Lys11-linked chain extension, and UBE2N, together with the catalytically inactive cofactors UBE2V1 or UBE2V2, supports Lys63-linked chain assembly (47–51). More broadly, E2 enzymes can partition ubiquitination into mechanistically distinct initiation and elongation phases, thereby shaping the architecture and downstream consequences of the ubiquitin signal (51–57).

The UBC fold also contains partially overlapping interaction surfaces for E1 and E3 binding, ensuring that E1-E2 and E2-E3 engagement are mutually exclusive during ubiquitin transfer (58, 59). This organization underlies the catalytic logic of E3 cooperation. RING and U-box ligases promote direct ubiquitin transfer from E2~Ub to substrate, whereas HECT and RBR ligases first form an E3~Ub thioester intermediate before substrate modification (60–62). Consistent with this mechanistic distinction, RING/U-box E3s generally stabilize a closed E2~Ub conformation, while HECT and RBR ligases do not uniformly require this state (35, 63–68). Beyond the canonical E3-binding interface, the E2 “backside” has emerged as an additional regulatory surface that modulates processivity, allostery, and ubiquitin signal specification through non-covalent interactions with ubiquitin or E3-associated elements (69–75). Collectively, these observations establish that UBE2 enzymes actively determine transfer chemistry, linkage specificity, and signaling output. In the following sections, we therefore examine, in sequence, how distinct UBE2 family members contribute to cancer-associated phenotypes, including proliferative control, oncogenic signaling, metabolic adaptation, immune evasion, therapeutic resistance, and biomarker potential. The detailed mechanistic and translational analyses that follow, an at-a-glance categorization of UBE2 family members according to their predominant oncogenic versus tumor-suppressive roles across cancer lineages is provided in **Table 1**.

**Table 1:** Summary categorization of UBE2 family members by predominant oncogenic versus tumor-suppressive roles across human cancers.

UBE2 enzyme	Oncogenic context (cancer type/key mechanism)	Tumor-suppressive context (cancer type/key mechanism)	Functional class	Key references
Dual/context-dependent UBE2 family members (oncogenic in some lineages, tumor-suppressive in others)				
UBE2C	TNBC, melanoma, LUAD, AML, hepatoblastoma, HCC, breast, PDAC, HNSCC, ovarian, gastric (intestinal-type), CCA, prostate, UCEC, ESCC; pan-cancer (33 tumor types)	Gastric (ferroptosis-prone context); thyroid (loss enhances migration, invasion, and sorafenib resistance)	Dual/context-dependent (predominantly oncogenic; context-dependent vulnerability)	(76, 77, 80, 82, 83, 88, 92–94, 113, 117, 124, 128, 137, 145–147, 149, 150, 156, 165, 167, 168)
UBE2D3	PDAC (TAP2/MHC-I antigen-presentation blockade); glioma (SHP-2/STAT3 axis); ccRCC (3-gene prognostic model)	Esophageal cancer (downregulated in tumor; hTERT-inverse; independent favorable prognostic factor)	Dual/context-dependent	(22, 105, 124, 152)
UBE2J1	HGSOC (plasma-cell marker; promotes EMT and invasion)	CRC (promoter hypermethylation; TRIM25/RPS3-mediated NF-κB blockade); prostate (AR ubiquitination/degradation; loss promotes antiandrogen resistance)	Dual/context-dependent	(81, 134, 151)
UBE2M	CRC (neddylation of USP39/PABPC1/CCNB1 axis); ER+ breast (ERα stabilization driving fulvestrant resistance)	Melanoma (MKK7 neddylation → JNK/EGR1 axis restraining proliferation)	Dual/context-dependent	(21, 78, 133)
UBE2S	LUAD, HCC, gastric, NSCLC, ovarian, endometrial, GBM, osteosarcoma, SKCM, ESCC, breast; pan-cancer	CRC (TRAF6 ubiquitination → coordinate suppression of PI3K/AKT and MAPK/ERK)	Dual/context-dependent	(25, 28, 90, 101, 103, 106–109, 119, 127, 131, 132, 153, 154, 164, 175)
Predominantly oncogenic UBE2 family members (no tumor-suppressive context)				
UBE2B	Gastric (K63-TRAF1 ubiquitination, NF-κB feedforward loop); ESCA (NF-κB/EMT/inflammatory pathways)	Not reported	Predominantly oncogenic	(102, 155)
UBE2D1	GBM (STUB1-mediated p21 ubiquitination, G1/S override)	Not reported	Predominantly oncogenic	(86)
UBE2D2	Gastric peritoneal metastasis (autophagy-dependent CST1/GPx4 stabilization, ferroptosis suppression)	Not reported	Predominantly oncogenic	(118)
UBE2F	Lung adenocarcinoma (Cullin5 neddylation, NOXA degradation, platinum resistance); KRAS-mutant PDAC (CRL5^ASB11^–DIRAS2 axis, MAPK–c-Myc signaling)	Not reported	Predominantly oncogenic	(129, 170, 173)
UBE2H	PDAC (UBE2H+ ductal subpopulation, comprehensive immune suppression); LUAD (hypoxia-mediated metastasis)	Not reported	Predominantly oncogenic	(123, 148)
UBE2I (UBC9)	Thyroid (SUMOylated hnRNPA2B1, M2 polarization); ovarian (TAM remodeling, PD-L1 axis); cholangiocarcinoma (SUMOylation-driven p27 nuclear export)	Not reported	Predominantly oncogenic	(121, 122, 135)
UBE2K	HCC (c-Myc upregulation; HIF-1α axis under hypoxia); breast (STUB1 degradation, PKA/CREB1 positive feedback)	Not reported	Predominantly oncogenic	(84, 99, 112)
UBE2L3	TNBC (SMURF2-mediated TSC2 degradation; mTOR maintenance; autophagy and anti–PD-1 sensitization upon loss)	Not reported	Predominantly oncogenic	(136)
UBE2L6	Cisplatin-resistant solid tumors (ABCB6 transporter upregulation)	Not reported	Predominantly oncogenic	(125)
UBE2N	Melanoma (MEK/FRA1/SOX10 cascade); prostate (Axin1 ubiquitination, Wnt/β-catenin, glycolysis); LUAD (chemoresistance and metastasis); AML (oncogenic innate immune signaling)	Not reported	Predominantly oncogenic	(85, 114, 138, 139, 141)
UBE2O	Breast and prostate (AMPKα2 degradation → mTOR/HIF-1α axis); breast cancer stem cells (UBE2O/AMPKα2/mTORC1–MYC feedback loop)	Not reported	Predominantly oncogenic	(87, 140)
UBE2Q2	HCC (K63-RIP1 ubiquitination, NF-κB-driven HIF-1α upregulation, glycolytic rewiring)	Not reported	Predominantly oncogenic	(111)
UBE2T	CRC, HCC, breast, LUAD, PDAC, ESCC, multiple myeloma (Wnt/β-catenin, Akt/β-catenin, pyrimidine biosynthesis, autophagy, FA pathway, glycolysis/PD-L1)	Not reported	Predominantly oncogenic	(23, 79, 89, 91, 95, 100, 115, 116, 120, 130, 143, 144, 157, 163, 166)
UBE2V1	HCC (HIF-1α–driven positive feedback; competitive VHL binding and K11/K48 VHL ubiquitination with UBE2S)	Not reported	Predominantly oncogenic	(110)

## 3 Cell cycle dysregulation and proliferative control by UBE2 enzymes

Uncontrolled proliferation requires systematic subversion of cell cycle checkpoints that normally restrain mitotic progression. The ubiquitin–proteasome system plays an indispensable role in this regulation through the timely degradation of cyclins, cyclin-dependent kinase inhibitors, and checkpoint mediators. Several UBE2 family members, most notably UBE2C, UBE2S, and UBE2T, function as essential cofactors of the anaphase-promoting complex/cyclosome (APC/C), and their aberrant overexpression disrupts this coordinated degradation program, enabling checkpoint override and resistance to apoptotic signals. Here, we examine how these enzymes drive proliferative control through three interconnected mechanisms: direct regulation of mitotic progression, disruption of the p53/p21 tumor-suppressor axis, and engagement of context-specific signaling effectors across hematologic and solid tumors (**Table 2**).

**Table 2:** UBE2 enzymes in cell cycle control, apoptotic evasion, and context-dependent across proliferative signaling human cancers.

UBE2 enzyme	Cancer type	Role	Key substrate/pathway	Functional outcome	Validation	Ref.
UBE2C/S/T as master drivers of mitotic progression						
UBE2C	TNBC	Oncogene	Cyclins/cell cycle checkpoints; DNA repair pathways; apoptosis pathways (GSEA)	Accelerates G1/S and G2/M transitions (P<0.01); reduces DNA damage accumulation (P<0.01); suppresses apoptosis (P<0.01); high expression correlates with reduced OS (P = 0.01)	TCGA/GTEx; MDA-MB-231 (qRT-PCR, WB, flow cytometry, comet, TUNEL)	(76)
UBE2C	Melanoma	Oncogene	MPF (mitosis-promoting factor); ERK/Akt signaling pathways	Silencing blocks G2/M transition via MPF downregulation; deactivates ERK/Akt; triggers apoptosis; high expression correlates with poor OS (p<0.01)	TCGA; fresh melanoma samples; RNA interference; xenograft nude mice *in vivo*	(77)
UBE2M	CRC	Oncogene	USP39 (neddylation substrate) → PABPC1 (deubiquitination) → CCNB1 (translation)	Neddylation of USP39 modulates PABPC1 deubiquitination, enhances CCNB1 translation, propels G2/M progression	Single-cell + bulk transcriptome; *in vivo* + *in vitro*; micafungin identified as UBE2M inhibitor	(78)
UBE2C/S/T	ER+ Breast	Therapeutic target	APC/C complex; G1/S progression; ubiquitin-proteasome pathway	CDK4/6 inhibitors (palbociclib, ribociclib) suppress UBE2C/S/T expression (mRNA + protein); not shared with abemaciclib; UBE2C/UBE2T levels associated with survival	HR+ cell lines MCF7, T47D; biochemical assays; clinical database cohorts	(79)
p53/p21 axis disruption and apoptosis evasion						
UBE2C	LUAD	Oncogene	p53 (ubiquitin-dependent degradation) → p53/p21 signaling inactivation	Promotes p53 ubiquitination and degradation; suppresses p53 and p21 levels; enables proliferation, migration, invasion; rescue experiments confirm p53/p21 dependency	TCGA; qRT-PCR, WB; nude mice xenograft *in vivo*	(80)
UBE2T	CRC	Oncogene	p53 (ubiquitination and degradation)	Facilitates p53 ubiquitination; correlates with adverse clinicopathological features and reduced OS (P = 0.0455); promotes growth, proliferation, invasion, inhibits apoptosis	qRT-PCR, IHC, Kaplan-Meier; subcutaneous CRC model *in vivo*	(23)
UBE2S	Gastric	Oncogene	p53 (ubiquitination inhibition upon KO) → FAS upregulation → extrinsic apoptotic pathway	CRISPR/Cas9 KO suppresses growth *in vitro* and *in vivo*; RNA-seq + TMT proteomics reveal FAS-mediated apoptosis; negative UBE2S–FAS correlation in tissues	CRISPR/Cas9; multi-omics (RNA-seq + TMT proteomics); *in vivo*	(25)
UBE2J1	CRC	TSG	RPS3 (ubiquitination at K214, with TRIM25) → NF-κB nuclear translocation blockade	Downregulated via promoter hypermethylation; forms E2–E3 complex with TRIM25; ubiquitinates RPS3; inactivates NF-κB; inhibits proliferation and metastasis	qRT-PCR, WB, IHC; *in vitro* + *in vivo*	(81)
Context-specific proliferative roles across hematologic and solid tumors						
UBE2C	AML	Oncogene	PI3K/AKT signaling pathway; ferroptosis regulation	Overexpressed in AML; activates PI3K/AKT; KD inhibits viability, promotes apoptosis, increases Fe2+ and ROS, enhances erastin-induced ferroptosis (proteasome-dependent)	TCGA; HL60, THP-1, U937, KG-1 cells; xenograft mouse model	(82)
UBE2C	Hepatoblastoma	Oncogene	Cell cycle regulation (RNA-seq after KD)	Upregulated in 5/6 HB lines; IHC: 20/25 tumors vs. 1/6 normal; silencing decreases viability; correlates with inferior survival	RNA-seq (5 PDX + 1 cell line); IHC validation (25 HB, 6 normal)	(83)
UBE2K	HCC	Oncogene	c-Myc (upregulation; functional rescue confirms dependency)	Elevated in HCC cells; promotes proliferation, migration, stemness; KD suppresses malignant phenotypes; UBE2K/c-Myc axis validated by rescue experiments	RT-qPCR, WB, CCK-8, Transwell, sphere formation; *in vivo*	(84)
UBE2N	Melanoma	Oncogene	MEK/ERK → FRA1/SOX10 signaling cascade; K63-specific ubiquitin conjugase	Silencing decreases proliferation; E-cadherin↑, p16↑; SOX10↓, Nestin↓; active FRA1 restores growth; phosphoproteomics confirms MEK/ERK impairment	Mass spectrometry phosphoproteomics; NSC697923 (UBE2N inhibitor) reduces xenograft growth *in vivo*	(85)
UBE2M	Melanoma	TSG	MKK7 (neddylation substrate) → JNK → EGR1 → CCND2 suppression	Downregulated in melanoma tissues; neddylation of MKK7 inhibits its ubiquitination, stabilizes MKK7, enhances phosphorylation; activates JNK/EGR1; restrains proliferation	WB, IF, Co-IP, CHX chase, PLA; *in vitro* + *in vivo*	(21)
UBE2D1	GBM	Oncogene	p21 (ubiquitination and degradation via STUB1 E3 ligase)	Highly expressed; KD inhibits growth and causes G1 arrest; UBCH5A binds p21 at protein level; STUB1-mediated negative regulation of p21	qRT-PCR, WB, functional assays	(86)
UBE2O	Breast/Prostate	Oncogene	AMPKα2 (ubiquitination and degradation) → mTOR–HIF1α axis activation	Ube2o deletion in MMTV-PyVT/TRAMP mice impairs tumor initiation, growth, metastasis; switches off metabolic reprogramming; AMPKα2 (not α1) mediates effect; rapamycin/HIF1α inhibition ablates UBE2O biology	Ube2o KO mice (MMTV-PyVT, TRAMP); pharmacological blockade restores AMPKα2 *in vivo*	(87)

### 3.1 UBE2C/S/T as master drivers of mitotic progression

Faithful mitotic progression requires the timely ubiquitin-dependent degradation of cyclins and checkpoint regulators, a process in which several UBE2 enzymes play central roles. UBE2C has emerged as a particularly prominent mitotic driver across multiple tumor types. In triple-negative breast cancer (TNBC), UBE2C overexpression accelerates both G1/S and G2/M transitions while simultaneously suppressing DNA damage accumulation and apoptosis, and its elevated expression correlates with reduced overall survival (76). A concordant role has been described in melanoma, where UBE2C silencing blocks G2/M progression through downregulation of the mitosis-promoting factor (MPF) and concurrent inactivation of ERK/AKT signaling, effects validated in xenograft models (77). Beyond canonical ubiquitination, the neddylation-specific conjugating enzyme UBE2M links NEDD8 modification to cell cycle control in colorectal cancer by neddylating USP39, which stabilizes PABPC1 and enhances CCNB1 translation, thereby propelling G2/M progression (78). Importantly, the therapeutic relevance of this axis is underscored by evidence that CDK4/6 inhibitors, palbociclib and ribociclib, suppress UBE2C, UBE2S, and UBE2T expression in estrogen receptor-positive breast cancer, suggesting that part of their clinical efficacy may derive from disruption of the ubiquitin–proteasome pathway (79). Collectively, these findings suggest that UBE2C/S/T occupy an important position at the intersection of mitotic control and oncogenic signaling, while highlighting shared vulnerabilities and context-dependent regulatory mechanisms across diverse malignancies.

### 3.2 p53/p21 axis disruption and apoptosis evasion

The p53/p21 tumor-suppressor axis constitutes a critical checkpoint governing proliferative arrest and apoptotic commitment, and its inactivation through ubiquitin-dependent mechanisms represents a recurring theme in UBE2-driven oncogenesis. In lung adenocarcinoma (LUAD), UBE2C directly promotes p53 ubiquitination and proteasomal degradation, thereby suppressing both p53 and p21 protein levels and enabling unchecked proliferation, migration, and invasion; notably, rescue experiments confirmed that the malignant phenotypes induced by UBE2C are contingent upon functional inactivation of the p53/p21 pathway (80). A mechanistically parallel role has been attributed to UBE2T in colorectal cancer (CRC), where its overexpression facilitates p53 ubiquitination and degradation, correlates with adverse clinicopathological features and reduced overall survival, and drives tumor growth both *in vitro* and in subcutaneous xenograft models (23). These convergent findings suggest that p53 ubiquitination by distinct E2 enzymes may constitute a recurrent mechanism of apoptosis evasion across epithelial malignancies.

Beyond direct p53 targeting, UBE2S engages the p53 axis through an indirect mechanism in gastric cancer. CRISPR/Cas9-mediated knockout of UBE2S inhibits p53 ubiquitination and degradation, leading to FAS upregulation and activation of the extrinsic apoptotic pathway, as demonstrated by integrated transcriptomic and proteomic analyses (25). In contrast, UBE2J1 exerts a tumor-suppressive function in CRC: its promoter is silenced by DNA hypermethylation, and restored UBE2J1 expression forms an E2–E3 complex with TRIM25 to ubiquitinate RPS3, thereby blocking NF-κB nuclear translocation and restraining proliferation and metastasis (81). This opposing directionality underscores the functional diversity within the UBE2 family, wherein individual members may either dismantle or reinforce p53-dependent tumor suppression depending on substrate specificity and cellular context.

### 3.3 Context-specific proliferative roles across hematologic and solid tumors

The proliferative functions of UBE2 enzymes extend well beyond the canonical mitotic roles discussed above, with individual family members engaging distinct signaling effectors in a tissue- and context-dependent manner. In acute myeloid leukemia (AML), UBE2C overexpression activates the PI3K/AKT pathway to sustain proliferation and simultaneously suppress ferroptosis; UBE2C knockdown increases intracellular Fe²^+^ and reactive oxygen species levels, sensitizing AML cells to erastin-induced ferroptotic death (82). In pediatric hepatoblastoma, UBE2C is markedly upregulated relative to normal hepatocytes, correlates with inferior patient survival, and its silencing decreases cell viability with concomitant alterations in cell cycle gene expression (83). Similarly, in hepatocellular carcinoma (HCC), UBE2K drives proliferation, migration, and stemness through upregulation of c-Myc, establishing the UBE2K/c-Myc axis as a functionally validated oncogenic module (84).

In melanoma, UBE2N and its heterodimeric partners UBE2V1/V2 sustain tumor growth via the MEK/FRA1/SOX10 signaling cascade, and systemic delivery of the small-molecule UBE2N inhibitor NSC697923 significantly reduces xenograft growth, providing early proof-of-concept for pharmacological UBE2 targeting (85). Intriguingly, UBE2M exhibits an opposing, tumor-suppressive role in melanoma: its downregulation impairs neddylation of MKK7, destabilizing MKK7 and attenuating JNK/EGR1 signaling, thereby relieving proliferative restraint (21). This dichotomy between UBE2N-mediated oncogenic signaling and UBE2M-mediated tumor suppression within the same malignancy highlights the functional heterogeneity of the E2 enzyme family.

In glioblastoma, UBE2D1 promotes proliferation by partnering with the E3 ligase STUB1 to ubiquitinate p21, inducing G1/S checkpoint override (86). At the interface of metabolism and proliferation, UBE2O targets AMPKα2 for degradation, activating the mTOR–HIF1α axis to drive tumor initiation, growth, and metabolic reprogramming in breast and prostate cancer models; pharmacological blockade of UBE2O restores AMPKα2 and suppresses tumorigenesis *in vivo* (87). Collectively, these findings indicate that UBE2 enzymes influence diverse oncogenic and metabolic pathways sustaining proliferation. Their net effect, oncogenic or tumor-suppressive, is shaped by substrate specificity and the signaling architecture of each tumor type.

Beyond their direct effects on cell-cycle progression and genomic stability, the proliferation-associated UBE2 enzymes discussed above, most notably UBE2C, UBE2T, and UBE2S, exert downstream effects with documented immunomodulatory consequences. UBE2C activity has been linked to PD-L1 regulation through PI3K/AKT signaling and to tumor-mutational-burden landscapes across pan-cancer cohorts (88); UBE2T-driven pyrimidine biosynthesis and replication stress alter the antigenic substrate available for MHC-I presentation (89); and UBE2S sustains a JAK2/STAT3-permissive transcriptional program with documented effects on tumor-associated macrophage infiltration (90). The proliferation-centered findings reviewed in this section are therefore not immunologically inert: they constitute the cell-intrinsic substrate upon which the tumor–immune crosstalk reviewed later in this manuscript ultimately operates.

## 4 Oncogenic signaling pathways orchestrated by UBE2 family members

Beyond their roles in cell cycle control and apoptosis evasion described in Section 1, UBE2 enzymes serve as critical amplifiers of oncogenic signaling cascades that sustain tumor growth, metabolic adaptation, and metastatic progression. The PI3K/AKT/mTOR, MAPK/ERK, Wnt/β-catenin, Notch, NF-κB, and STAT pathways represent the most frequently co-opted axes. Multiple UBE2 family members converge on these shared signaling nodes through mechanistically distinct upstream routes, a pattern that suggests both functional redundancy and potential combinatorial therapeutic vulnerability (**Table 3**).

**Table 3:** Oncogenic signaling pathways orchestrated by UBE2 family members across human cancers.

UBE2 enzyme	Cancer type	Signaling pathway	Key substrate/mechanism	Functional outcome	Validation	Ref.
PI3K/AKT/mTOR and MAPK/ERK pathway amplification						
UBE2C	HCC	PI3K/AKT/mTOR	Facilitates PTEN ubiquitination and degradation; activates PI3K/AKT/mTOR; upregulates MTHFD2 via ATF4-mediated transcription	Maintains redox homeostasis; elevates PD-L1 expression; links proliferative signaling to immune evasion; UBE2C-MTHFD2 axis inhibition suppresses HCC *in vivo*	*In vitro* + *in vivo*; clinical correlation (UBE2C/MTHFD2 co-expression)	(94)
UBE2T	HCC	MAPK/ERK → Wnt/β-catenin	Promotes β-catenin nuclear translocation through MAPK/ERK-dependent activation; disrupts E-cadherin/β-catenin membrane complex	EMT induction; E-cadherin loss with cytoplasmic/nuclear β-catenin elevation; MAPK/ERK inhibition (not AKT/mTOR) blocks β-catenin translocation	HCC cell lines; chemical inhibition of MAPK/ERK, AKT/mTOR, Wnt pathways	(95)
UBE2K	Breast	PKA/CREB1	Ubiquitinates and degrades STUB1; activates PKA/CREB1 pathway; CREB1 transcriptionally activates UBE2K (positive feedback loop)	Promotes proliferation and migration; UBE2K/CREB1 feedback loop sustains malignant progression	RT-qPCR, Transwell, WB, CCK-8; ChIP and dual-luciferase confirm CREB1 as UBE2K transcription factor	(99)
UBE2C	Breast	MAPK	Transcriptionally upregulated by MAZ; UBE2C activates MAPK signaling pathway	Promotes proliferation, migration, invasion; MAZ/UBE2C/MAPK regulatory circuit drives breast cancer progression *in vivo*	IHC, RT-qPCR, WB; CCK-8, Transwell, cloning; ChIP; nude mouse xenograft	(97)
UBE2S	Ovarian	PI3K/AKT/mTOR	Knockdown inhibits PI3K/AKT/mTOR signaling pathway	Blocks cell cycle progression; promotes apoptosis; inhibits proliferation, invasion, and migration (P<0.05)	TCGA, GEPIA, GEO; SKOV3, A2780 cells; IHC, Kaplan-Meier (n=181); functional assays	(101)
UBE2C	Breast	AKT/mTOR	Knockdown increases p-PTEN (P<0.050); decreases p-AKT, p-mTOR, and HIF-1α (P<0.050)	Inhibits proliferation and invasion; UBE2C maintains AKT/mTOR axis	GEO + TCGA DEGs; PPI network; RT-qPCR, CCK-8, Transwell, WB	(96)
UBE2C	Breast	JNK	Modulates JNK signaling pathway; affects EMT markers and proliferation/apoptosis proteins	Promotes proliferation, migration, invasion, metastasis; suppresses apoptosis; KD suppresses transplanted tumor growth in mice	TCGA + GEO; *in vitro* assays; subcutaneous tumor model; IHC (E-cadherin, Ki-67, p53)	(98)
UBE2T	Breast	PI3K/AKT	Regulates PI3K/AKT signaling pathway; affects EMT and glycolytic proteins	Promotes proliferation, invasion, glycolysis (ATP, glucose consumption, lactate production); KD suppresses tumor growth in mice	qRT-PCR, WB; CCK-8, colony formation, Transwell; xenograft; IHC (UBE2T, Ki-67)	(100)
Wnt/β-catenin and notch signaling						
UBE2T	HCC	Wnt/β-catenin	Ubiquitinates E3 ligase Mule; prevents Mule-mediated β-catenin degradation; E2 activity required (abolished when impaired)	Enhances liver CSC self-renewal, drug resistance, tumorigenicity, metastasis; UBE2T/Mule/β-catenin cascade regulates liver CSCs	Integrative CSC datasets; *in vitro* + *in vivo*; Co-IP confirms Mule as binding partner	(91)
UBE2C	HCC	Notch	Activates Notch signaling; upregulates N1ICD and Hes1; activates RBP-JK luciferase reporter	Promotes growth and metastasis; oncogenic role eliminated by DAPT (Notch inhibitor); N1ICD overexpression rescues UBE2C-KD effects	TCGA + clinical specimens; *in vitro*; rescue experiments with DAPT and N1ICD	(92)
UBE3C	Gastric	Wnt/β-catenin	Ubiquitinates and degrades AXIN1 (destruction-complex scaffold); activates β-catenin signaling	Promotes proliferation; inhibits apoptosis; KD increases AXIN1 and reduces nuclear/cytoplasmic β-catenin in xenograft tissues	TCGA RNA-seq; *in vitro* (KD + overexpression); nude mouse model *in vivo*	(176)
UBE2C	PDAC	MAPK (via KRAS stabilization)	Cooperates with APC/C^CDH1^ to degrade WDR76 (KEN-box dependent); WDR76 normally targets WT/mutant KRAS for CUL1-mediated degradation; KRAS accumulation activates MAPK	Ube2c deletion suppresses tumorigenesis/metastasis in KrasG12D and KrasG12D;p53-/- mice; AAV-mediated WDR76 KD fully rescues Ube2c deletion phenotypes	Cell culture + KrasG12D GEM models; AAV pancreatic duct injection; clinical PDAC tissues	(93)
*NF-κB and STAT Signaling Axes*						
UBE2B	Gastric	NF-κB	Interacts with E3 ligase BIRC2; catalyzes K63-linked ubiquitination of TRAF1; P65 binds UBE2B promoter (feedforward loop)	Amplifies NF-κB signaling; self-sustaining UBE2B-BIRC2-TRAF1 axis; promotes proliferation *in vitro* and *in vivo*	Bioinformatics + clinical validation; ChIP + luciferase (P65→UBE2B promoter)	(102)
UBE2S	LUAD	NF-κB	Directly binds IκBα independently of canonical IKK phosphorylation; promotes IκBα degradation and nuclear P65 accumulation	Activates NF-κB signaling; promotes EMT and metastasis; IKK inhibitors (PS1145, SC514) do not alter IκBα phosphorylation	GST pull-down, *in vitro* binding, IF; luciferase; zebrafish xenograft metastasis model	(103)
UBE2C	Pancreatic	PI3K-Akt (via EGFR)	Binds and stabilizes EGFR; drives PI3K-Akt pathway activation; promotes MMP expression	Promotes metastatic progression and glycolytic activity (glucose uptake, lactate, ATP); KD impairs glycolysis	qRT-PCR; glycolysis kits; mechanistic studies	(104)
UBE2D3	Glioma	STAT3	Ubiquitinates and activates SHP-2; elevated SHP-2 enhances STAT3 phosphorylation; UBE2D3/SHP-2 show inverse expression patterns	Promotes proliferation and glycolysis; inhibition reverses phenotypes *in vitro* and *in vivo* (xenograft)	TCGA-GBM; ECAR/OCR measurement; xenograft *in vivo*	(105)
UBE2S	HCC	VHL/HIF-1α and VHL/JAK2/STAT3	Directly interacts with VHL; upregulates HIF-1α and JAK2/STAT3 signaling; downregulation enhances sorafenib sensitivity	Promotes proliferation and migration via dual VHL/HIF-1α and VHL/JAK2/STAT3 axes; reduces sorafenib sensitivity	WB, qRT-PCR, IHC, IF, siRNA, Co-IP; animal models *in vivo*	(106)
UBE2S	NSCLC	c-Myc (via RPL26)	Targets RPL26 for ubiquitination and degradation; RPL26 loss upregulates c-Myc	Promotes proliferation, migration, stemness; UBE2S inhibition suppresses RPL26-c-Myc-mediated tumor growth *in vivo*	IP-MS identifies RPL26 as substrate; xenograft *in vivo*	(108)
UBE2S	CRC	PI3K/AKT and MAPK/ERK (inhibition)	Promotes ubiquitin-mediated degradation of TRAF6 (upstream activator of both pathways); blocked by MG132	TSG: overexpression inhibits proliferation and G0/1→G2 progression; KD enhances proliferation; larger tumors upon KD *in vivo*	IHC; TCGA/GTEx (positive OS correlation); RNA-seq; HCT116/RKO; animal experiments	(107)

### 4.1 Wnt/β-catenin and notch signaling

Aberrant activation of Wnt/β-catenin signaling is a hallmark of multiple gastrointestinal and hepatic malignancies, and several UBE2 enzymes have been implicated in its regulation. In HCC, UBE2T promotes cancer stemness by ubiquitinating the E3 ligase Mule, thereby preventing Mule-mediated β-catenin degradation and sustaining Wnt pathway activation; this cascade enhances self-renewal, drug resistance, and metastatic capacity of liver cancer stem cells (91). UBE2 enzymes also intersect with Notch signaling. UBE2C activates the Notch pathway in HCC by upregulating cleaved Notch1 (N1ICD) and its transcriptional target Hes1, effects that are abolished by pharmacological Notch inhibition with DAPT (92). In pancreatic ductal adenocarcinoma (PDAC), UBE2C engages a distinct route by cooperating with the APC/C^CDH1^ E3 ligase to degrade WDR76, a CUL1-associated adaptor that normally targets both wild-type and mutant KRAS for proteasomal degradation; the resulting KRAS accumulation activates MAPK signaling and fuels tumorigenesis, as demonstrated in genetically engineered KrasG12D-driven mouse models (93). Collectively, these studies position UBE2 enzymes as versatile modulators of developmental signaling pathways co-opted during tumorigenesis, with therapeutic implications for both Wnt- and Notch-addicted cancers.

### 4.2 PI3K/AKT/mTOR and MAPK/ERK pathway amplification

The proliferative and metabolic advantages conferred by UBE2 enzymes are frequently channeled through the PI3K/AKT/mTOR and MAPK/ERK signaling axes, two of the most commonly hyperactivated cascades in human cancers. In HCC, UBE2C facilitates PTEN ubiquitination and degradation, thereby derepressing PI3K/AKT/mTOR signaling and upregulating the one-carbon metabolism enzyme MTHFD2 via ATF4-mediated transcription; this axis simultaneously maintains redox homeostasis and elevates PD-L1 expression, linking proliferative signaling to immune evasion (94). UBE2T further amplifies oncogenic signaling in HCC by promoting β-catenin nuclear translocation through MAPK/ERK-dependent activation, with pharmacological inhibition of MAPK/ERK, but not AKT/mTOR, being sufficient to block β-catenin nuclear accumulation and EMT (95).

**Figure 1:**
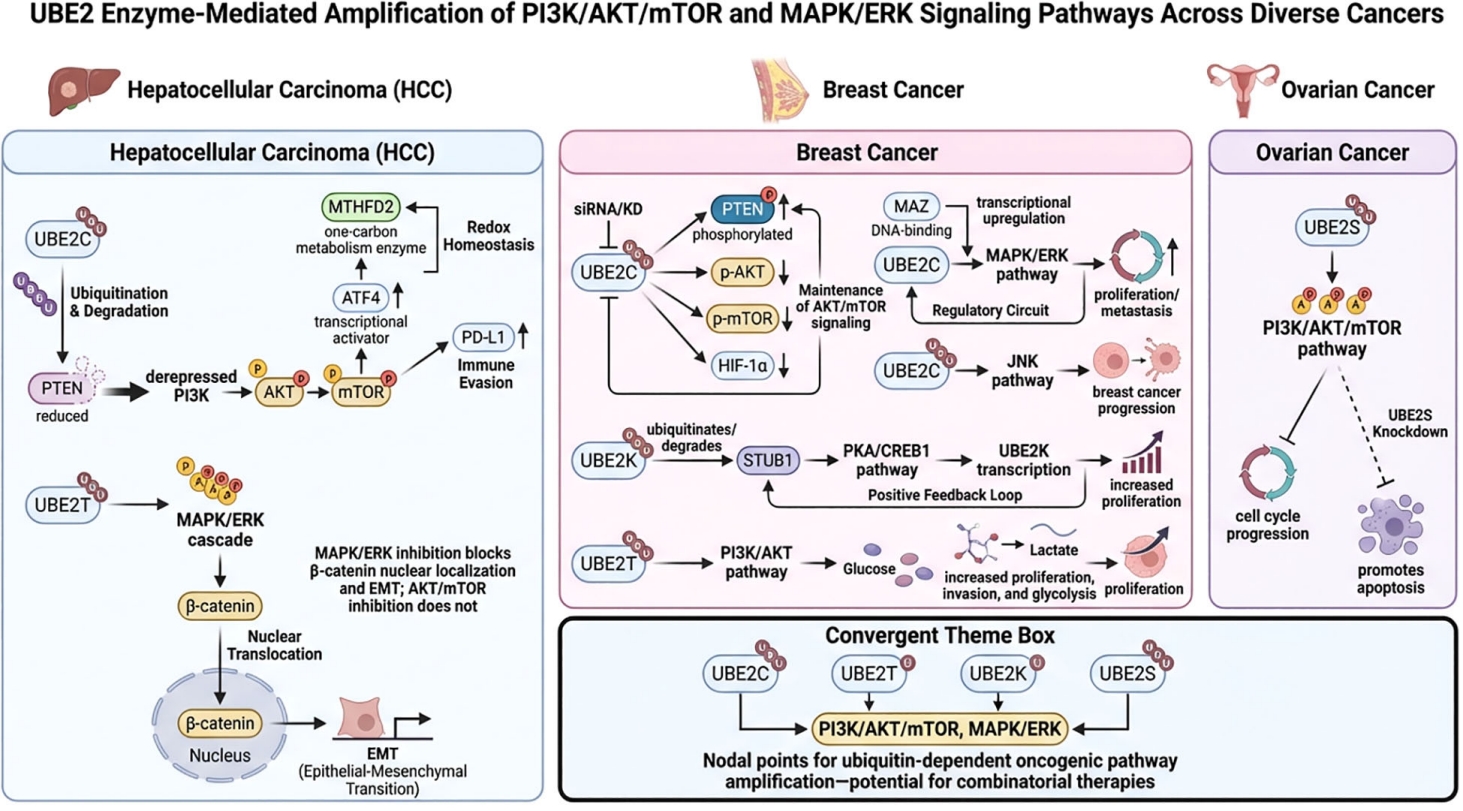
UBE2 enzyme-mediated amplification of PI3K/AKT/mTOR and MAPK/ERK signaling across diverse cancers. In HCC, breast cancer, and ovarian cancer, distinct UBE2 family members enhance oncogenic signaling by targeting regulators such as PTEN, STUB1, and β-catenin-associated pathways, thereby promoting proliferation, metabolic adaptation, immune evasion, invasion, and metastasis. These recurrent pathway interactions identify PI3K/AKT/mTOR and MAPK/ERK as convergent ubiquitin-dependent signaling hubs and support their potential as targets for combinatorial therapeutic intervention.

In breast cancer, multiple E2 enzymes converge on these pathways through distinct upstream mechanisms. UBE2C knockdown increases phosphorylated PTEN and decreases p-AKT, p-mTOR, and HIF-1α levels, directly implicating UBE2C in AKT/mTOR axis maintenance (96). The transcription factor MAZ transcriptionally upregulates UBE2C, which in turn activates MAPK signaling to drive proliferation and metastasis, establishing the MAZ/UBE2C/MAPK regulatory circuit (97). UBE2C also engages JNK signaling to promote breast cancer progression, highlighting pathway versatility within a single E2 enzyme (98). Independently, UBE2K ubiquitinates and degrades STUB1 to activate the PKA/CREB1 pathway, forming a positive feedback loop that sustains its own transcription and drives breast cancer growth (99). UBE2T similarly promotes breast cancer proliferation, invasion, and glycolysis through PI3K/AKT activation (100). Beyond breast and liver cancers, UBE2S activates the PI3K/AKT/mTOR cascade in ovarian cancer, where its knockdown blocks cell cycle progression and promotes apoptosis (101). The recurrent engagement of PI3K/AKT and MAPK/ERK by multiple UBE2 family members across diverse malignancies suggests that these pathways act as nodal points of convergent ubiquitin-dependent regulation and may offer opportunities for rational combinatorial targeting (**Figure 1**).

### 4.3 NF-κB and STAT signaling axes

In addition to the PI3K/AKT and MAPK cascades, UBE2 enzymes modulate NF-κB and STAT signaling, two pathways intimately linked to inflammation-driven tumor progression. In gastric cancer, UBE2B cooperates with the E3 ligase BIRC2 to catalyze K63-linked ubiquitination of TRAF1, amplifying NF-κB signaling; NF-κB subunit P65 in turn binds the UBE2B promoter to enhance its transcription, establishing a self-sustaining feedforward loop that maintains pathway hyperactivation (102). UBE2S engages NF-κB through an alternative mechanism in lung adenocarcinoma, where it directly binds IκBα independently of canonical IKK-mediated phosphorylation, promoting IκBα degradation, nuclear P65 accumulation, and EMT-associated metastasis validated in zebrafish xenograft models (103). UBE2C similarly activates PI3K-Akt signaling in pancreatic cancer through stabilization of EGFR, thereby promoting glycolytic activity and metastatic progression (104).

STAT signaling represents another convergence point for UBE2-mediated regulation. In glioma, UBE2D3 ubiquitinates and activates SHP-2, which enhances STAT3 phosphorylation to drive both proliferation and aerobic glycolysis; UBE2D3 inhibition reverses these phenotypes *in vivo* (105). In hepatocellular carcinoma, UBE2S interacts with VHL and concurrently activates both HIF-1α and JAK2/STAT3 signaling, promoting proliferation and migration while simultaneously reducing sensitivity to sorafenib, thereby linking STAT pathway activation to therapeutic resistance (106).

**Figure 2:**
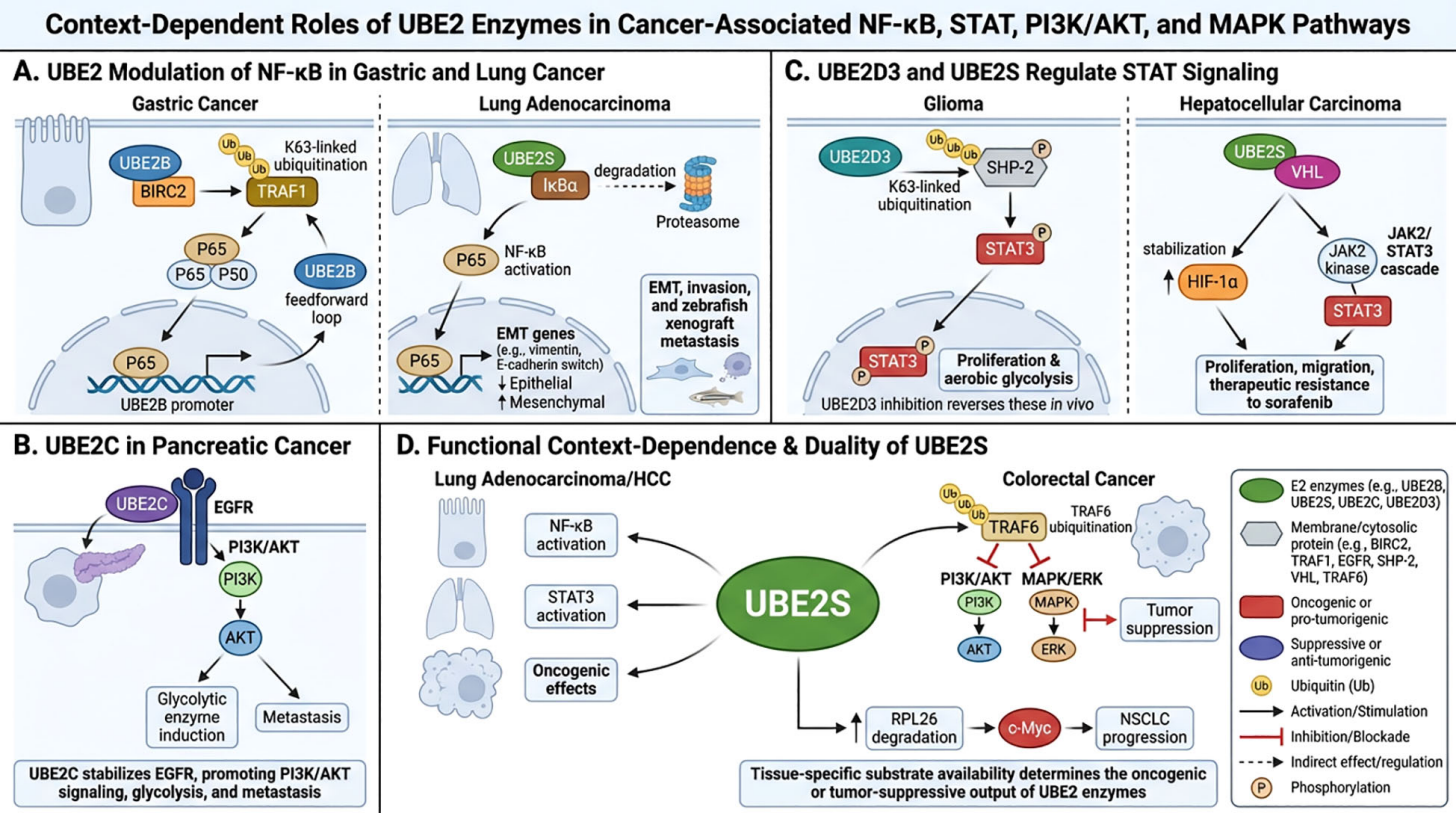
Context-dependent regulation of NF-κB, STAT, PI3K/AKT, and MAPK signaling by UBE2 enzymes across multiple cancers. **(A)** NF-κB activation via UBE2B/BIRC2-mediated K63-linked TRAF1 ubiquitination and a P65 feedforward loop in gastric cancer, and UBE2S-driven IκBα degradation promoting EMT, invasion, and metastasis in lung adenocarcinoma. **(B)** UBE2C-dependent EGFR stabilization activating PI3K/AKT to drive glycolytic enzyme induction and metastasis in pancreatic cancer. **(C)** STAT signaling through UBE2D3/SHP-2 in glioma and UBE2S/VHL-mediated HIF-1α stabilization with JAK2/STAT3 activation in hepatocellular carcinoma, linked to proliferation, migration, and sorafenib resistance. **(D)** Functional duality of UBE2S, which is oncogenic in lung adenocarcinoma, HCC, and NSCLC (via NF-κB, STAT3, and RPL26/c-Myc) but tumor-suppressive in colorectal cancer through TRAF6 ubiquitination that inhibits PI3K/AKT and MAPK/ERK, underscoring the role of tissue-specific substrate context in shaping UBE2-dependent outputs.

Notably, the functional valence of individual UBE2 enzymes within these pathways is context-dependent. Whereas UBE2S acts as an oncogene in lung adenocarcinoma and HCC through NF-κB and STAT3 activation (103, 106), it paradoxically functions as a tumor suppressor in colorectal cancer by ubiquitinating TRAF6 to simultaneously inhibit both PI3K/AKT and MAPK/ERK cascades (107). UBE2S also targets RPL26 for degradation to upregulate c-Myc and promote non-small cell lung cancer progression, illustrating how a single E2 enzyme can engage entirely distinct substrates across tumor types (108). This functional duality underscores the importance of tissue-specific substrate availability in determining the oncogenic or tumor-suppressive output of UBE2 signaling networks. Importantly, however, these opposing roles have been documented in independent studies using different cell systems and experimental endpoints, and direct comparative analyses, such as side-by-side substrate proteomics or isogenic knock-out models across tumor lineages, are not yet available. The mechanistic basis for this lineage dependence therefore remains inferential rather than experimentally established, a caveat we revisit below in the dedicated critical appraisal section (**Figure 2**).

It is important to note that the oncogenic signaling pathways modulated by UBE2 enzymes, PI3K/AKT, MAPK/ERK, NF-κB, JAK2/STAT3, and Wnt/β-catenin, are themselves principal regulators of antitumor immunity, rather than purely cell-intrinsic proliferation circuits. PI3K/AKT activation drives PD-L1 stabilization and metabolic reprogramming in immunosuppressive directions (94); NF-κB signaling shapes inflammatory cytokine output and macrophage polarization (103); JAK2/STAT3 governs MDSC accumulation and T-cell exclusion (106); and Wnt/β-catenin activation is a well-characterized mechanism of T-cell exclusion across multiple tumor types (91, 95). The signaling-level findings catalogued here therefore carry direct immunological implications, and several UBE2-driven signaling perturbations should be regarded simultaneously as proliferation-promoting and immunoediting events.

## 5 Metabolic reprogramming, hypoxia adaptation, and ferroptosis

The oncogenic signaling and metastatic programs driven by UBE2 enzymes, as described in the preceding sections, are intimately coupled to metabolic reprogramming that sustains the bioenergetic and biosynthetic demands of tumor cells. Rapidly proliferating cancer cells require not only enhanced glycolytic flux but also augmented nucleotide biosynthesis, redox homeostasis, and resistance to metabolic stress-induced cell death. Emerging evidence reveals that UBE2 enzymes play central roles in each of these metabolic adaptations. They achieve this primarily through convergent regulation of hypoxia-inducible factor 1α (HIF-1α) and its downstream effectors. UBE2 enzymes also modulate ferroptosis, an iron-dependent cell death pathway increasingly recognized as a therapeutic target (**Table 4**).

**Table 4:** UBE2 enzymes in metabolic reprogramming and tumor microenvironment remodeling.

UBE2 enzyme	Cancer type	Functional category	Key mechanism	Substrate/target	Downstream effect	Therapeutic implication	Ref
Glycolytic rewiring and HIF-1α regulation							
UBE2S	HCC	Glycolysis/HIF-1α	Enhances E3-independent K11-linked polyubiquitination of VHL at K171 and K196; promotes VHL proteasomal degradation	VHL	HIF-1α stabilization; glycolysis activation	Cephalomannine + PX-478 combination improves antitumor efficacy	(109)
UBE2V1	HCC	Glycolysis/HIF-1α	Transcriptionally activated by HIF-1α; competes with HIF-1α for VHL binding; catalyzes K11/K48-linked ubiquitination of VHL at K196 with UBE2S	VHL (K196)	Self-reinforcing HIF-1α–UBE2V1 positive feedback loop	UBE2V1 KD or HIF-1α inhibition suppresses HCC *in vivo*	(110)
UBE2Q2	HCC	Glycolysis/HIF-1α	Cooperates with cIAP1 to catalyze K63-linked ubiquitination of RIP1; activates NF-κB signaling	RIP1 (K63)	NF-κB–mediated transcriptional upregulation of HIF-1α; glycolysis↑	UBE2Q2 as therapeutic target for HCC	(111)
UBE2K	HCC	Glycolysis/HIF-1α	Hypoxia-responsive gene; transcriptionally induced by HIF-1α; depletion inhibits hypoxia-induced proliferation and migration	HIF-1α (upstream)	Functional HIF-1α/UBE2K axis	UBE2K depletion reverses hypoxia-driven phenotypes	(112)
UBE2C	HNSCC	Glycolysis/HIF-1α	Modulates HIF-1α expression and glycolysis pathway; associated with lymph node metastasis	HIF-1α/glycolytic enzymes	HIF-1α–dependent glycolytic flux; invasion/migration↑	CoCl2 restores glycolysis upon UBE2C KD; prognostic factor	(113)
UBE2N	Prostate	Glycolysis/Wnt	Promotes Axin1 ubiquitination; activates Wnt/β-catenin signaling	Axin1	Cell viability↑ and glycolysis↑	XAV939 or Axin1 overexpression reverses effects	(114)
Pyrimidine metabolism, autophagy, and immune evasion							
UBE2T	HCC	Pyrimidine metabolism	K63-linked ubiquitination of Akt; activates Akt/β-catenin; upregulates CAD, DHODH, UMPS	Akt (K63)	Pyrimidine metabolism↑; HCC progression	Leflunomide (DHODH inhibitor) blocks UBE2T-driven progression	(115)
UBE2T	LUAD	Autophagy	Downregulates p53; activates AMPK/mTOR; promotes autophagy	p53/AMPK/mTOR	Cisplatin resistance via autophagy; reversible upon inhibition	Prognostic signature with autophagy genes stratifies survival	(116)
Ferroptosis regulation							
UBE2C	Gastric	Ferroptosis suppression	Ubiquitinates and degrades ACSL4; reduces ROS and iron accumulation	ACSL4	Ferroptosis inhibition; GSH rescues *in vivo*	Targeting UBE2C/ACSL4 to induce ferroptosis	(117)
UBE2D2	Gastric (peritoneal met)	Ferroptosis suppression	Autophagy-dependent stabilization of CST1; preserves GPx4 levels	CST1/GPx4	Ferroptosis inhibition; KD induces ROS-mediated ferroptosis, suppresses peritoneal metastasis	UBE2D2–CST1–GPx4 as therapeutic target	(118)
UBE2S	Endometrial	Ferroptosis suppression	KD simultaneously induces cell cycle arrest, apoptosis↑, and ferroptosis↑	Multiple	Suppresses multiple cell death modalities; immunosuppressive TME	UBE2S as ferroptosis induction target	(119)
Antigen presentation and CD8^+^ T cell suppression							
UBE2D3	PDAC	Antigen presentation blockade	IFN-γ-driven overexpression; cooperates with KLHL13; K63-polyubiquitination of TAP2 at K245 blocks peptide transport	TAP2 (K245)	MHC-I↓; CD8^+^ T surveillance impaired	UBE2D3 inhibition + KRASG12D TCR-T: synergistic	(22)
UBE2T	LUAD	PD-L1/CD8^+^ T suppression	FOXA1 upregulates UBE2T; activates glycolysis; elevates PD-L1	Glycolysis/PD-L1	CD8^+^ T cytotoxicity↓; KD enhances T cell infiltration *in vivo*	2-DG reverses immunosuppressive phenotype	(120)
UBE2I	Thyroid	M2 macrophage polarization	SUMOylation of hnRNPA2B1; cytoplasmic relocalization; M2 polarization	hnRNPA2B1 (SUMO)	M2 TAM↑; CD206↑ *in vivo*	SUMOylation inhibition reverses effects	(121)
Macrophage polarization and microenvironment remodeling							
UBE2I	Ovarian	Macrophage reprogramming	Depletion reprograms TAMs M2→M1 via glycolysis↑ (glucose consumption↑, lactate↑, ECAR↑); glycolysis inhibitor reverses dose-dependently	TAM metabolism	M1↑; PD-L1↑ on M1; apoptosis↑	UBE2I inhibitor + anti-PD-L1: superior efficacy *in vivo*	(122)
UBE2H	PDAC	Comprehensive immune suppression	UBE2H^+^ ductal population (scRNA-seq); enhances PD-L1 axis; impairs antigen presentation (H2Kb, I-Ab↓)	T cells, macrophages, neutrophils	T exhaustion (PD-1↑, CD69↓, GZMB↓); NET↑; cold tumor	Target for cold→hot tumor conversion	(123)
UBE2S	Pan-cancer	TAM infiltration	Overexpressed; associated with TAM infiltration across tumor types	TAMs	Adverse features; poor prognosis; conserved mechanism	Pan-cancer predictor of macrophage immunosuppression	(90)
Immune checkpoint regulation and Treg induction							
UBE2C	Pan-cancer (33 types)	Immune modulation (broad)	Correlates with TMB in 20 cancers; advanced stage in 8; GSEA: immune pathways	Multiple immune pathways	Immune response modulation	Diagnostic and immunotherapy-predictive biomarker	(88)
UBE2C, UBE2D3, UBE2T	ccRCC	Treg induction	3-gene model (AUC 0.745/0.766/0.771); UBE2C KD reduces TGF-β1, decreasing Treg ratio	TGF-β1/Tregs	Treg induction; immune tolerance; high-risk: TMB↑, immunotherapy↓	Prognosis + immunotherapy + chemo prediction	(124)

### 5.1 Glycolytic rewiring and HIF-1α regulation

A central theme emerging from recent studies is the convergent regulation of hypoxia-inducible factor 1α (HIF-1α) by multiple UBE2 family members, which in turn drives aerobic glycolysis across diverse malignancies. In HCC, this convergence is particularly striking: UBE2S enhances E3 ligase-independent K11-linked polyubiquitination of VHL at residues K171 and K196, promoting VHL proteasomal degradation and indirectly stabilizing HIF-1α to drive glycolysis; combination of the UBE2S inhibitor cephalomannine with the HIF-1α inhibitor PX-478 significantly improves antitumor efficacy (109). UBE2V1 reinforces this axis through a distinct mechanism, competing with HIF-1α for VHL binding and catalyzing K11/K48-linked ubiquitination of VHL at K196 in complex with UBE2S, establishing a self-reinforcing HIF-1α–UBE2V1 positive feedback loop (110). UBE2Q2 activates glycolysis in HCC through an alternative route: it cooperates with cIAP1 to catalyze K63-linked ubiquitination of RIP1, activating NF-κB signaling, which transcriptionally upregulates HIF-1α (111). Furthermore, UBE2K is transcriptionally induced by HIF-1α under hypoxic conditions and reciprocally promotes HCC cell proliferation and migration, forming a functional HIF-1α/UBE2K axis (112). This glycolytic rewiring extends beyond HCC. In head and neck squamous cell carcinoma, UBE2C activates HIF-1α-dependent glycolytic flux, and CoCl_2_-mediated HIF-1α restoration rescues glycolytic enzyme expression and migratory capacity following UBE2C knockdown (113). In prostate cancer, UBE2N promotes glycolysis by ubiquitinating Axin1 and activating Wnt/β-catenin signaling; Axin1 overexpression or pharmacological Wnt inhibition with XAV939 reverses UBE2N-driven metabolic effects (114).

**Figure 3:**
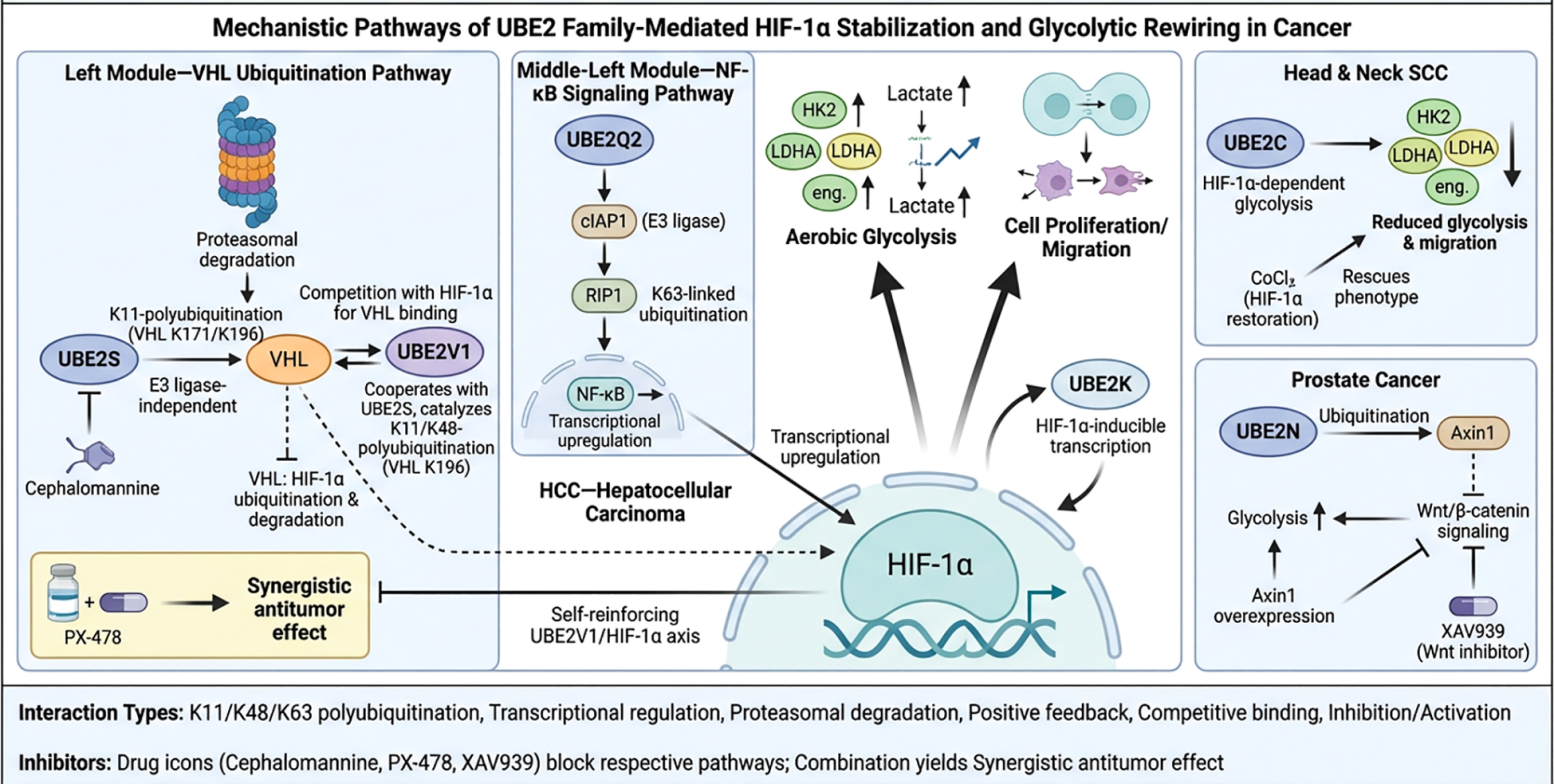
UBE2 family members promote glycolytic rewiring through convergent stabilization and activation of HIF-1α across multiple cancer types. In HCC, UBE2S, UBE2V1, and UBE2Q2 enhance HIF-1α signaling through distinct mechanisms including VHL ubiquitination and degradation, competitive VHL binding, and NF-κB–mediated transcriptional upregulation, while HIF-1α-induced UBE2K establishes a reciprocal pro-glycolytic circuit that supports proliferation and migration. This metabolic program extends to head and neck squamous cell carcinoma and prostate cancer through UBE2C- and UBE2N-dependent pathways, highlighting the HIF-1α/glycolysis axis as a convergent and therapeutically actionable node for combinatorial intervention.

Collectively, these findings suggest that UBE2 enzymes can converge on HIF-1α stabilization through at least three mechanistically distinct routes: direct VHL ubiquitination, competitive VHL binding, and NF-κB–mediated transcriptional activation. It is important to note, however, that this convergence has been most extensively characterized in hepatocellular carcinoma, with supporting but considerably less detailed evidence in head and neck squamous cell carcinoma and prostate cancer; whether the same hierarchy of mechanisms operates in tumors with intrinsically different oxygen-sensing biology, such as kidney cancers with constitutive VHL loss or hematologic malignancies with distinct hypoxic responses, remains an open question. With these tumor-context boundaries acknowledged, the HIF-1α/glycolysis axis emerges as a plausible target for combination-based therapeutic intervention in the lineages where the mechanism has been validated, while extrapolation to other tumor types should await direct experimental confirmation (**Figure 3**).

### 5.2 Pyrimidine metabolism, autophagy, and immune evasion

Beyond glycolytic rewiring, UBE2 enzymes reprogram nucleotide biosynthesis, autophagic flux, and immune microenvironment dynamics to sustain tumor growth. In hepatocellular carcinoma, UBE2T catalyzes K63-linked ubiquitination of Akt, activating the Akt/β-catenin cascade and upregulating the *de novo* pyrimidine synthesis enzymes CAD, DHODH, and UMPS; importantly, the clinically approved DHODH inhibitor leflunomide, originally developed for rheumatoid arthritis, effectively blocks UBE2T-driven HCC progression in preclinical models, raising the possibility of repurposing pending dedicated oncology trials with HCC-relevant patient selection and pharmacodynamic monitoring (115). In lung adenocarcinoma, UBE2T promotes autophagy through downregulation of p53 and subsequent activation of the AMPK/mTOR pathway. This UBE2T-induced autophagy confers cisplatin resistance that is reversible upon pharmacological autophagy inhibition, establishing a mechanistic link between metabolic adaptation and chemoresistance; a multigene prognostic signature incorporating UBE2T-associated autophagy genes significantly stratifies patient survival outcomes (116). These findings collectively demonstrate that UBE2-mediated metabolic reprogramming extends well beyond bioenergetic adaptation to encompass nucleotide supply, autophagic survival signaling, and immunosuppressive microenvironment remodeling.

### 5.3 Ferroptosis regulation

Emerging evidence identifies UBE2 enzymes as critical regulators of ferroptosis, an iron-dependent form of regulated cell death increasingly recognized as a tumor-suppressive mechanism. In gastric cancer, two distinct E2 enzymes converge on ferroptosis suppression through mechanistically complementary substrates. UBE2C directly ubiquitinates and degrades ACSL4, a key ferroptosis executor, thereby reducing reactive oxygen species (ROS) accumulation and intracellular iron levels; notably, GSH treatment *in vivo* alleviates the tumor-promoting effects of UBE2C overexpression, confirming the ferroptosis-dependent nature of this oncogenic axis (117). UBE2D2 operates through an alternative route, promoting autophagy-dependent stabilization of CST1, which in turn preserves GPx4 protein levels, the central enzymatic gatekeeper against ferroptotic lipid peroxidation; UBE2D2 knockdown destabilizes this protective cascade, inducing ROS-mediated ferroptosis and effectively suppressing peritoneal metastasis in preclinical models (118).

Beyond gastrointestinal malignancies, UBE2S overexpression in endometrial cancer correlates with adverse patient prognosis and an immunosuppressive tumor microenvironment; its knockdown simultaneously induces cell cycle arrest, enhanced apoptosis, and increased ferroptosis, indicating that UBE2S suppresses multiple cell death modalities in parallel (119). These converging findings collectively support ferroptosis induction—through pharmacological targeting of UBE2C/ACSL4, UBE2D2/CST1/GPx4, or UBE2S—as a biologically rational hypothesis for overcoming apoptosis resistance in advanced and metastatic malignancies. We emphasize, however, that this strategy currently rests on cell line and xenograft data, that no UBE2-directed ferroptosis inducer has entered clinical evaluation, and that the therapeutic window of ferroptosis induction in normal tissues with high UBE2 expression remains uncharacterized.

The metabolic reprogramming events outlined in this section, glycolytic rewiring through HIF-1α stabilization, pyrimidine biosynthesis amplification, and ferroptosis modulation, are inseparable from the immune microenvironment in which they occur. Glycolytic UBE2 activity not only fuels tumor cell proliferation but also depletes glucose from the tumor interstitium, restricting effector T-cell function and promoting tumor-associated macrophage M2 polarization (109, 120); pyrimidine metabolism modulated by UBE2T influences both DNA damage signaling and antigen presentation efficiency (89); and UBE2-mediated ferroptosis suppression simultaneously shields tumor cells from oxidative cell death and from immunogenic-cell-death–dependent T-cell priming (117, 119). These observations directly motivate the immune-microenvironment analysis that follows.

## 6 Tumor microenvironment remodeling and immune evasion

The metabolic reprogramming orchestrated by UBE2 enzymes, as detailed in the preceding section, profoundly shapes the tumor microenvironment, particularly through modulation of antitumor immunity. It is increasingly appreciated that the ubiquitin system directly regulates key components of both innate and adaptive immune responses, including antigen processing and presentation, immune checkpoint ligand expression, macrophage polarization, and regulatory T cell induction. UBE2 enzymes are increasingly implicated in these immunomodulatory processes, with important implications for understanding immunotherapy resistance and informing rational combination strategies. We emphasize, however, that the immunological roles documented to date have been characterized predominantly in pancreatic, lung, ovarian, thyroid, hepatocellular, and renal carcinoma models, and that the extrapolation of any individual UBE2–immune mechanism beyond its originally reported tumor type should be regarded as hypothesis-generating until validated in lineage-matched independent studies.

### 6.1 Antigen presentation disruption and CD8^+^ T cell suppression

UBE2 enzymes are increasingly implicated in immune evasion through direct interference with antigen presentation machinery and T-cell effector function. In pancreatic ductal adenocarcinoma (PDAC), IFN-γ, normally a cornerstone of antitumor immunity, paradoxically drives UBE2D3 overexpression from the earliest stages of pancreatic intraepithelial neoplasia, independently of oncogenic KRAS status. UBE2D3 cooperates with the E3 ligase KLHL13 to catalyze K63-linked polyubiquitination of the peptide transporter TAP2 at K245, sterically blocking peptide loading onto MHC-I molecules and effectively camouflaging tumor cells from immune surveillance. Importantly, although this UBE2D3–TAP2 axis represents a compelling tumor-intrinsic mechanism of antigen-presentation suppression, the IFN-γ–driven UBE2D3 induction has been formally demonstrated only in pancreatic intraepithelial neoplasia and PDAC; whether analogous IFN-γ feedback circuits operate in immunologically distinct tumor settings, such as melanoma or microsatellite-instability-high colorectal cancer where IFN-γ signaling has well-characterized but mechanistically different consequences, has not been directly tested and represents a key open question (22).

In lung adenocarcinoma, immune evasion is mediated through a metabolic route: the transcription factor FOXA1 transcriptionally upregulates UBE2T, which activates glycolysis and consequentially elevates PD-L1 expression, thereby suppressing CD8^+^ T cell cytotoxicity. The glycolysis inhibitor 2-deoxy-D-glucose (2-DG) reverses this immunosuppressive phenotype, and UBE2T knockdown enhances intratumoral CD8^+^ T cell infiltration *in vivo*, linking glycolytic rewiring to checkpoint ligand regulation (120).

**Figure 4:**
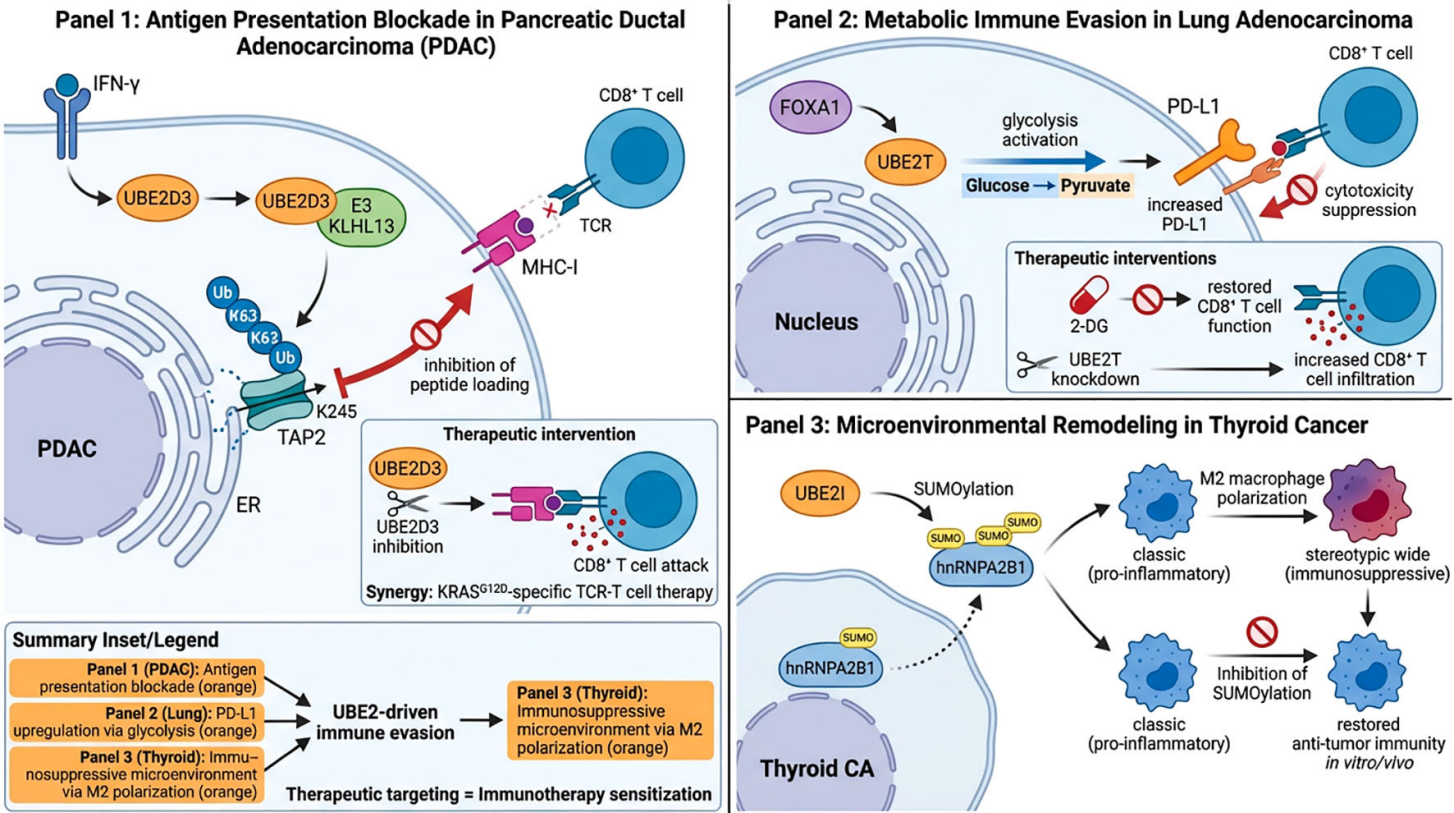
UBE2 enzymes promote immune evasion by disrupting antigen presentation, suppressing CD8^+^ T cell function, and remodeling the tumor microenvironment. In PDAC, UBE2D3 cooperates with KLHL13 to catalyze K63-linked ubiquitination of TAP2 and impair MHC-I peptide loading, whereas in lung adenocarcinoma FOXA1-driven UBE2T enhances glycolysis-dependent PD-L1 expression to inhibit CD8^+^ T cell cytotoxicity; in thyroid cancer, UBE2I promotes hnRNPA2B1 SUMOylation and M2 macrophage polarization. These mechanistically distinct but convergent immune-evasive programs are therapeutically actionable, as inhibition of UBE2D3, UBE2T-associated glycolysis, or UBE2I/SUMOylation restores antitumor immunity and supports immunotherapy sensitization.

Beyond direct T cell suppression, UBE2I contributes to immunosuppressive microenvironment remodeling in thyroid cancer by enhancing SUMOylation and cytoplasmic relocalization of hnRNPA2B1, which promotes M2 macrophage polarization; pharmacological inhibition of SUMOylation reverses UBE2I-mediated tumor-promoting effects both *in vitro* and *in vivo* (121). These findings collectively indicate that UBE2 enzymes can promote immune evasion through mechanistically diverse routes, including antigen-presentation blockade, glycolysis-driven PD-L1 upregulation, and macrophage reprogramming. Importantly, however, most of these mechanisms have been characterized in single tumor models with limited cross-validation in independent cohorts, and the relative quantitative contribution of each route to overall immune evasion remains unresolved. Whether targeting any single UBE2-dependent immune-evasion node will yield clinically meaningful benefit, or whether combinatorial intervention against multiple converging routes will be required, is a question that current evidence does not yet answer (**Figure 4**).

### 6.2 Macrophage polarization and immunosuppressive microenvironment remodeling

Complementing their roles in antigen presentation blockade and T cell suppression, UBE2 enzymes actively remodel the myeloid compartment of the tumor microenvironment to establish immunosuppressive niches. In ovarian cancer, UBE2I depletion reprograms tumor-associated macrophages (TAMs) from the immunosuppressive M2 phenotype toward the pro-inflammatory M1 state through enhanced glycolytic activity, as evidenced by increased glucose consumption, lactate production, and extracellular acidification rate in macrophages; this metabolic reprogramming is reversed by glycolysis inhibition in a dose-dependent manner. UBE2I-mediated M1 polarization also upregulates PD-L1 expression, providing a rationale for combination therapy; in preclinical models, concurrent UBE2I inhibition and anti-PD-L1 treatment produced greater antitumor effects than either agent alone, suppressing tumor growth and promoting apoptosis *in vivo* (122).

**Figure 5:**
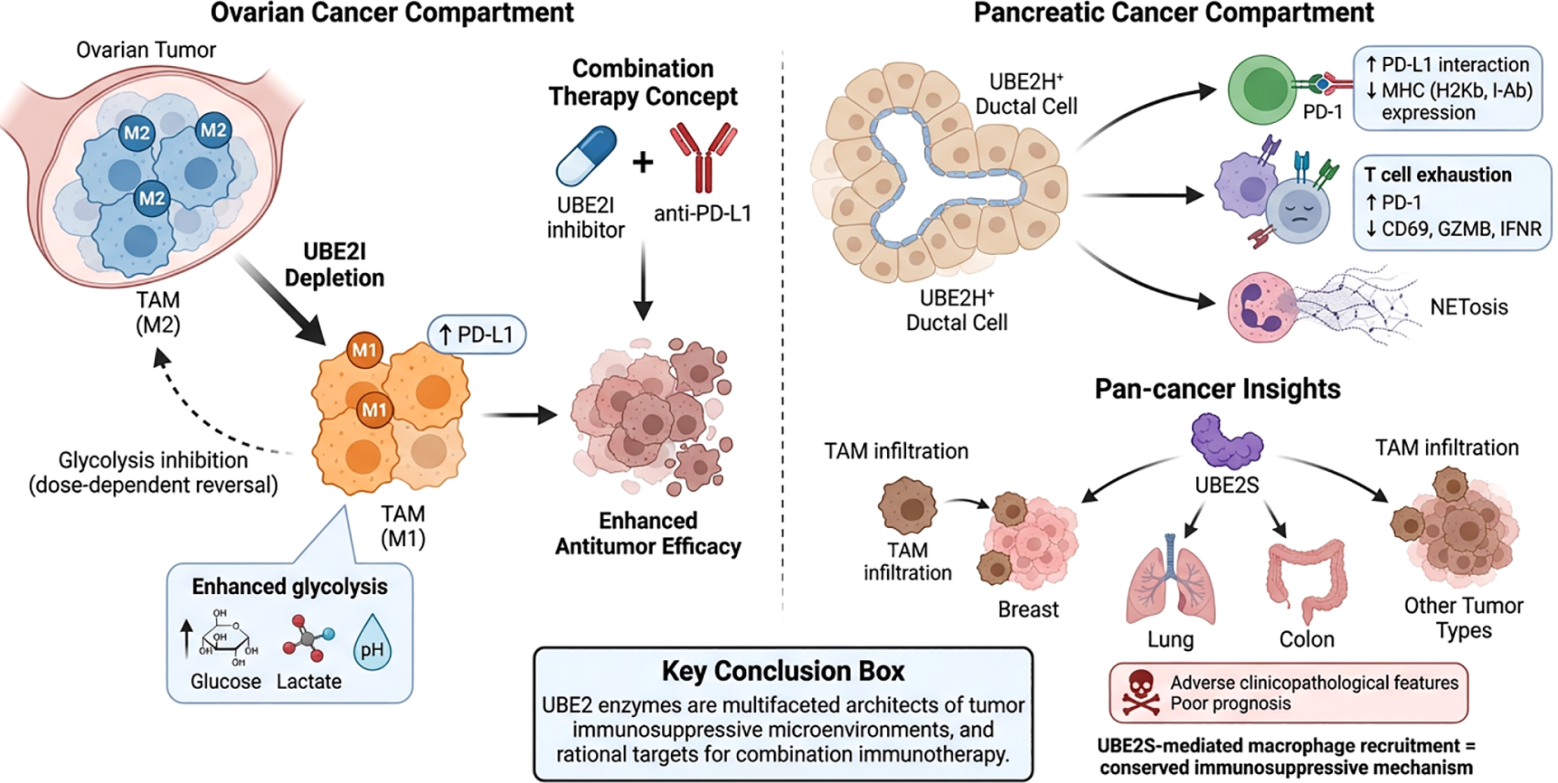
UBE2 enzymes remodel the myeloid compartment to establish immunosuppressive tumor microenvironments across ovarian, pancreatic, and multiple additional cancer types. UBE2I depletion drives glycolysis-dependent TAM repolarization from M2 to M1 in ovarian cancer and sensitizes tumors to combined anti-PD-L1 therapy, whereas UBE2H and UBE2S promote immune-cold phenotypes in pancreatic and pan-cancer settings through impaired antigen presentation, T cell exhaustion, NETosis, and macrophage infiltration.

In pancreatic cancer, a prototypical immunologically “cold” tumor, single-cell RNA sequencing has identified a distinct UBE2H^+^ malignant ductal cell population that comprehensively suppresses antitumor immunity. UBE2H overexpression simultaneously enhances the T cell PD-L1 interaction axis, impairs antigen presentation by macrophages and neutrophils through downregulation of MHC molecules (H2Kb, I-Ab), promotes T cell exhaustion marked by elevated PD-1 and diminished CD69, GZMB, and IFNR expression, and activates neutrophil extracellular trap (NET) formation (123). At the pan-cancer level, UBE2S overexpression is significantly associated with TAM infiltration across multiple tumor types, correlating with adverse clinicopathological features and poor prognosis, suggesting that UBE2S-mediated macrophage recruitment represents a conserved immunosuppressive mechanism (90). These converging findings support a role for UBE2 enzymes in shaping immunosuppressive microenvironments and suggest their relevance as targets in combination immunotherapeutic strategies (**Figure 5**).

### 6.3 Immune checkpoint regulation and regulatory T cell induction

UBE2 enzymes also modulate the efficacy of immune checkpoint blockade (ICB) through direct regulation of checkpoint ligands and immunosuppressive cell populations. Pan-cancer analyses have further implicated UBE2C as a broad immunomodulator with significant clinical relevance. In a comprehensive study encompassing 33 cancer types, Huang et al. demonstrated that UBE2C is differentially expressed across a wide spectrum of malignancies and that its elevated expression correlates with tumor mutational burden (TMB) in 20 cancer types (88). Notably, higher UBE2C expression was associated with advanced clinical stage in eight distinct malignancies, suggesting a link between UBE2C upregulation and tumor progression. Gene set enrichment analysis (GSEA) and immune relevance profiling consistently revealed that UBE2C participates in immune response pathways across multiple tumor contexts, reinforcing its potential utility as both a diagnostic indicator and an immunotherapeutic biomarker (88). These findings position UBE2C at the intersection of tumor biology and immune regulation, warranting deeper investigation into its mechanistic contributions to immune evasion.

Building on this pan-cancer perspective, Feng et al. provided more granular mechanistic insights in clear cell renal cell carcinoma (ccRCC) (124). By integrating bulk and single-cell RNA-sequencing data, the authors showed that UBE2 activity is significantly upregulated in malignant cells. Using machine learning, they constructed a three-gene prognostic model comprising UBE2C, UBE2D3, and UBE2T. This model exhibited robust predictive accuracy for overall survival, with AUC values of 0.745, 0.766, and 0.771 at 1, 3, and 5 years, respectively. Importantly, patients in the high-risk group showed poor responsiveness to immunotherapy. At the functional level, UBE2C knockdown in ccRCC cell lines reduced TGF-β1 secretion, which in turn decreased regulatory T cell (Treg) induction in co-culture systems (124). This observation directly links E2 enzyme activity to adaptive immune tolerance through the immunosuppressive Treg compartment. Collectively, these studies indicate that UBE2 enzymes regulate both innate and adaptive immune compartments. This dual role reinforces their potential as combinatorial targets for improving ICB efficacy across multiple tumor types (**Figure 5**).

## 7 Drug resistance and therapy sensitization mechanisms

The diverse oncogenic functions of UBE2 enzymes described in the preceding sections, spanning cell cycle control, signaling amplification, metabolic reprogramming, and immune evasion, converge to shape therapeutic responsiveness across multiple treatment modalities (**Table 5**).

**Table 5:** UBE2 enzymes in drug resistance, mechanisms and therapeutic reversal strategies.

UBE2 enzyme	Cancer type	Drug resistance	Resistance mechanism	Reversal strategy	Preclinical evidence	Ref.
Resistance to platinum-based chemotherapy						
UBE2L6	Multiple (cisplatin-R)	Cisplatin	Upregulated in resistant cells; transcriptionally regulates ABCB6	UBE2L6 silencing sensitizes resistant cells; overexpression alone insufficient to confer resistance	*In vitro*	(125)
RCN2/UBR5	ESCC	Cisplatin	RCN2 recruits UBR5 to ubiquitinate/degrade PPP2CA (PP2A), activating PI3K-AKT	RCN2 suppression synergizes with cisplatin	Subcutaneous + lung metastasis models	(126)
UBE2S	Ovarian	Cisplatin	Activates PI3K/AKT/mTOR; inhibits autophagy; higher in platinum-R vs. sensitive (IHC)	UBE2S KD restores autophagic flux and cisplatin sensitivity; mTOR agonist rescue	Resistant lines + xenograft *in vivo*	(127)
UBE2C	Ovarian	Cisplatin	Cooperates with CDK1 (strong co-expression); blocks G2/M upon depletion	UBE2C depletion reverses resistance via CDK1↓ and G2/M arrest	SKOV3/DDP, A2780/DDP; *in vitro* + *in vivo*	(128)
UBE2F	Lung adenocarcinoma	Platinum	Platinum disrupts UBE2F-RBX1 interaction; accumulated UBE2F enhances Cullin5 neddylation; NOXA↓	UBE2F knockout restores NOXA-dependent apoptosis	*In vitro* + *in vivo*	(129)
Resistance to cytotoxic and targeted agents						
UBE2T	CRC	Oxaliplatin, 5-FU	Upregulated in OXA/5-FU-R cells; activates Wnt/β-catenin in ERK-dependent manner	UBE2T KO restores sensitivity; re-expression rescues resistance (causal)	*In vitro* + xenograft *in vivo*	(130)
UBE2S	HCC	5-FU, Oxaliplatin	FOXM1→UBE2S→PTEN ubiquitination (K60/K327)→AKT phosphorylation	MK2206 (AKT inhibitor) attenuates chemoresistance	*In vitro*; independent risk factor OS/DFS	(131)
UBE2S	GBM	Temozolomide	Interacts with OTUB2; prevents K48 ubiquitination/degradation of PGAM1; enhances DNA repair	UBE2S KD enhances TMZ sensitivity	GBM mouse model *in vivo*	(132)
UBE2M	ER+ Breast	Fulvestrant	UBE2M-ERα feedback loop: ERα→HIF-1α→UBE2M transcription; UBE2M→CUL3/4A-E6AP→ERα stability	UBE2M silencing restores fulvestrant sensitivity	*In vitro* + *in vivo*	(133)
UBE2J1	Prostate	Antiandrogens	Critical E2 for AR ubiquitination/degradation; loss (5–15% patients) causes AR accumulation	Ubiquitination-based AR degrader restores degradation	Antiandrogen-resistant tumors *in vivo*	(134)
UBE2C/S/T	ER+ Breast	CDK4/6i (mechanism of efficacy)	Palbociclib/ribociclib suppress UBE2C/S/T (not abemaciclib); modulate APC/C and G1/S	UPP disruption contributes to CDK4/6i clinical efficacy	MCF7, T47D; clinical database cohorts	(79)
UBE2I	CCA	Chemotherapy	SUMOylation-dependent p27kip1 nuclear export via CRM1; cytoplasmic p27→chemoresistance	UBE2I silencing: p27 nuclear retention, cell cycle arrest, chemosensitivity↑	*In vitro* + xenograft *in vivo*	(135)
Sensitization: immunotherapy and combination approaches						
UBE2L3	TNBC	Anti-PD-1 (sensitization)	Cooperates with SMURF2 to degrade TSC2; sustains mTOR; suppresses autophagy	UBE2L3 KD: TSC2↑, autophagy↑, CD8^+^ T↑, potentiates anti-PD-1	*In vivo* CRISPR/Cas9 screen + combination	(136)
UBE2C	Breast	Doxorubicin (sensitization)	FOXM1-driven; maintains TOP2A stability	UBE2C inhibition: Parkin/K63-ubiquitination of TOP2A→senescence→doxorubicin sensitivity↑	*In vitro*	(137)
UBE2N	LUAD	Cisplatin (sensitization)	Promotes chemoresistance and metastasis	Wilforine (natural UBE2N inhibitor) reverses resistance + suppresses metastasis	*In vitro* + *in vivo*	(138)
UBE2N	AML	Immune signaling (sensitization)	Maintains oncogenic innate immune signaling; ubiquitinates inflammatory substrates	UC-764864/65 (active-site inhibitors): selective leukemic stem cell death, spares normal HSPCs	In silico screen + *in vitro*/*in vivo* + patient samples	(139)
UBE2O	Breast	Stemness-driven resistance	UBE2O/AMPKα2/mTORC1-MYC feedback loop; MYC transcriptionally promotes UBE2O	UBE2O KD suppresses tumor growth and lung metastasis	MDA-MB-231 xenograft *in vivo*	(140)

### 7.1 Resistance to platinum-based chemotherapy

Platinum compounds remain foundational in the treatment of numerous solid malignancies, yet resistance mediated by UBE2 enzymes constitutes an emerging and therapeutically actionable barrier. In cisplatin-resistant cells, UBE2L6 is significantly upregulated and sustains resistance by transcriptionally regulating the ABC transporter ABCB6; UBE2L6 silencing sensitizes resistant cells to cisplatin, whereas its overexpression alone is insufficient to confer resistance, suggesting that UBE2L6 maintains rather than initiates the resistant phenotype (125). In esophageal squamous cell carcinoma, the calcium-binding protein RCN2 recruits the E3 ligase UBR5 to ubiquitinate and degrade PPP2CA, a PP2A catalytic subunit, thereby activating PI3K-AKT signaling and conferring both metastatic capacity and cisplatin resistance; targeted RCN2 suppression synergizes with cisplatin in subcutaneous and lung metastasis models (126).

In ovarian cancer, platinum resistance is driven by at least two distinct UBE2 enzymes. UBE2S activates the PI3K/AKT/mTOR axis to suppress autophagy, with immunohistochemical analyses confirming significantly higher UBE2S expression in platinum-resistant versus platinum-sensitive clinical specimens; UBE2S knockdown restores autophagic flux and cisplatin sensitivity in resistant cell lines and xenograft models (127). UBE2C depletion similarly reverses cisplatin resistance in ovarian cancer through CDK1 downregulation and G2/M arrest, with strong co-expression of UBE2C and CDK1 observed in patient tissues (128).

**Figure 6:**
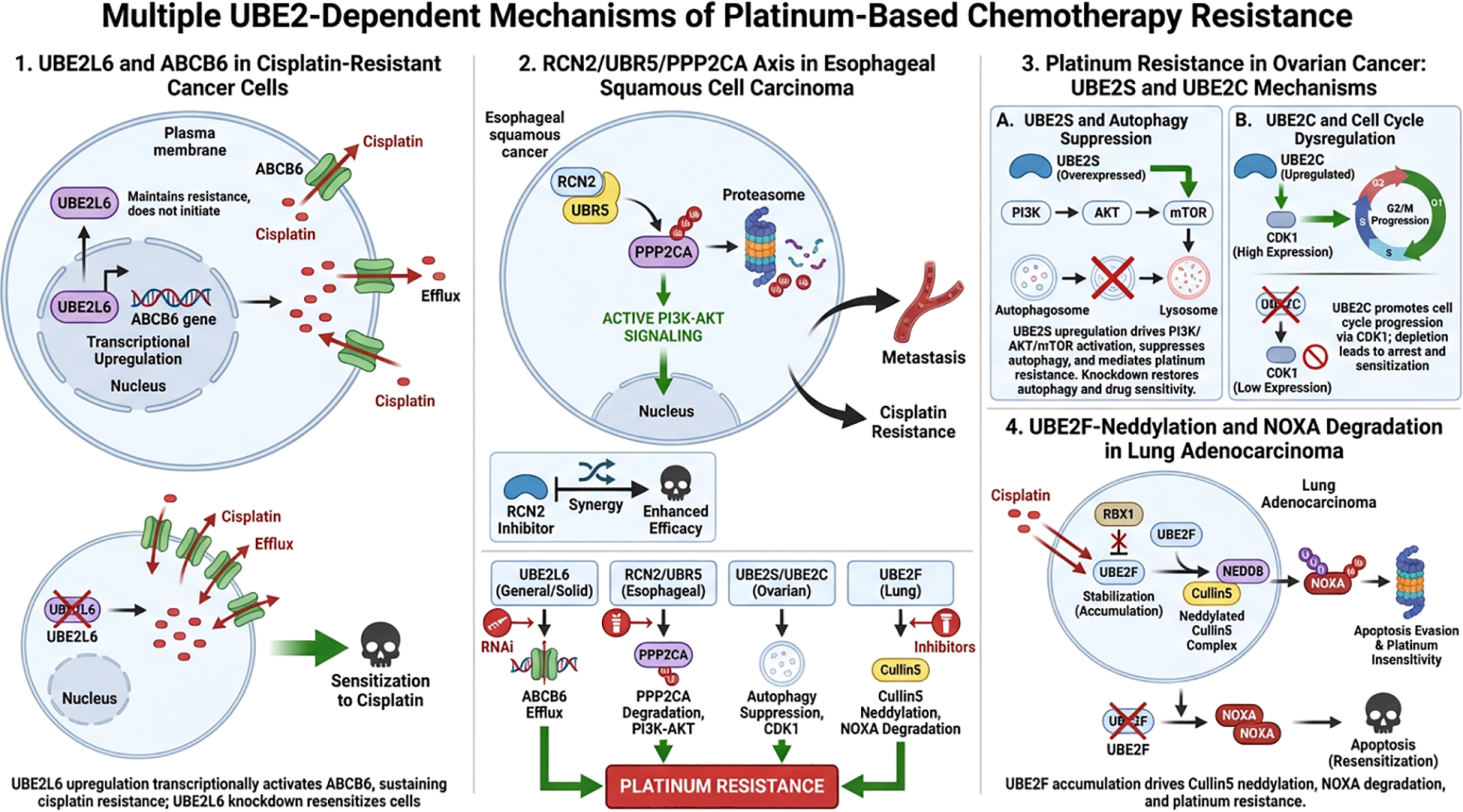
UBE2-dependent mechanisms of platinum-based chemotherapy resistance across multiple solid tumors. UBE2 family members sustain cisplatin or platinum resistance through distinct pathways, including UBE2L6-driven ABCB6 upregulation, RCN2/UBR5-mediated PPP2CA degradation and PI3K-AKT activation, UBE2S-dependent autophagy suppression, UBE2C-associated CDK1-driven cell cycle progression, and UBE2F-mediated Cullin5 neddylation with NOXA degradation, highlighting multiple actionable nodes for therapeutic resensitization.

Beyond canonical ubiquitination, the neddylation-specific E2 enzyme UBE2F mediates platinum insensitivity in lung adenocarcinoma. Platinum treatment paradoxically stabilizes UBE2F by disrupting its interaction with RBX1, reducing proteasomal degradation; accumulated UBE2F enhances Cullin5 neddylation, promoting degradation of the pro-apoptotic protein NOXA. UBE2F knockout significantly sensitizes lung cancer cells to platinum by restoring NOXA-dependent apoptosis (129). These findings collectively indicate that platinum resistance can arise through multiple UBE2-dependent mechanisms, including transporter regulation, autophagy suppression, cell-cycle dysregulation, and neddylation-mediated apoptosis evasion. It must be noted, however, that the majority of these mechanisms have been documented in established cell lines under single-drug selection pressure, and translation to patient-derived models with intact tumor heterogeneity and microenvironment remains limited. The clinical relevance of any individual UBE2-mediated resistance route therefore awaits validation in prospective cohorts before therapeutic prioritization can be confidently established (**Figure 6**).

### 7.2 Resistance to cytotoxic and targeted agents

Beyond platinum-based regimens, UBE2 enzymes confer resistance to diverse cytotoxic and targeted therapies through pathway-specific mechanisms. In colorectal cancer, UBE2T is significantly upregulated in oxaliplatin (OXA)- and 5-fluorouracil (5-FU)-resistant cells, where it activates Wnt/β-catenin signaling in an ERK-dependent manner; UBE2T knockout restores chemosensitivity both *in vitro* and in xenograft models, while UBE2T re-expression rescues the resistant phenotype, establishing a causal relationship (130). In hepatocellular carcinoma, FOXM1 transcriptionally activates UBE2S, which promotes PTEN ubiquitination at K60 and K327, driving AKT phosphorylation and resistance to 5-FU and oxaliplatin; the allosteric AKT inhibitor MK2206 attenuates this FOXM1–UBE2S-driven chemoresistance (131). UBE2S similarly mediates temozolomide (TMZ) resistance in glioblastoma through a mechanistically distinct route: it interacts with the deubiquitinase OTUB2 to prevent K48-linked ubiquitination and degradation of PGAM1, thereby stabilizing PGAM1, enhancing DNA repair, and reducing TMZ-induced apoptosis (132).

Resistance to endocrine and targeted therapies is similarly governed by UBE2 enzymes through hormone receptor stabilization. In ER-positive breast cancer, UBE2M and ERα form a positive feedback loop: ERα enhances HIF-1α-mediated transcription of UBE2M, while UBE2M maintains ERα stability by inhibiting its ubiquitination through the UBE2M–CUL3/4A–E6AP axis; UBE2M silencing restores fulvestrant sensitivity both *in vitro* and *in vivo* (133). In prostate cancer, UBE2J1 serves as a critical E2 enzyme directing AR ubiquitination and degradation; UBE2J1 loss, observed in 5–15% of patients, disrupts AR turnover, promotes AR protein accumulation, and fosters antiandrogen resistance, which can be overcome by ubiquitination-based AR degraders (134).

CDK4/6 inhibitors, palbociclib and ribociclib, suppress UBE2C, UBE2S, and UBE2T expression in ER-positive breast cancer, suggesting that disruption of the ubiquitin–proteasome pathway contributes to their clinical efficacy beyond cell cycle inhibition (79). In cholangiocarcinoma, UBE2I mediates SUMOylation-dependent nuclear export of the tumor suppressor p27kip1 via CRM1; UBE2I silencing induces p27kip1 nuclear retention, cell cycle arrest, and enhanced chemosensitivity, identifying UBE2I-mediated SUMOylation as a novel resistance mechanism (135). These findings collectively demonstrate that UBE2 enzymes engage multiple resistance-promoting pathways, Wnt/β-catenin, AKT, DNA repair, hormone receptor stabilization, and SUMOylation, providing a mechanistic rationale for combinatorial strategies pairing UBE2-targeted interventions with standard-of-care therapies.

### 7.3 Sensitization strategies: immunotherapy and combination approaches

The resistance mechanisms mediated by UBE2 enzymes, as outlined above, simultaneously reveal opportunities for therapeutic sensitization through targeted intervention. *In vivo* CRISPR/Cas9 library screening in TNBC identified UBE2L3 as a critical regulator whose loss restricts tumor growth by modulating autophagic flux: UBE2L3 cooperates with the E3 ligase SMURF2 to ubiquitinate and degrade TSC2, thereby sustaining mTOR activity and suppressing autophagy. UBE2L3 knockdown increases TSC2 expression, promotes autophagic cell death, enhances intratumoral CD8^+^ T-cell infiltration, and significantly potentiates anti-PD-1 therapy, establishing UBE2L3 as a dual target for tumor-intrinsic growth inhibition and immunotherapy sensitization (136).

UBE2C inhibition offers a complementary sensitization approach in breast cancer. FOXM1 transcriptionally drives UBE2C overexpression, and UBE2C inhibition promotes Parkin-mediated K63-linked ubiquitination of topoisomerase IIα (TOP2A), leading to its proteasomal degradation, cellular senescence, and markedly enhanced sensitivity to doxorubicin (137). In lung adenocarcinoma, UBE2N drives both chemoresistance and metastasis; the natural compound wilforine functions as a UBE2N inhibitor, effectively reversing cisplatin resistance and suppressing metastasis *in vivo* (138). In acute myeloid leukemia, structure-based screening identified two small-molecule UBE2N inhibitors (UC-764864/65) that block oncogenic innate immune signaling by disrupting ubiquitination of inflammatory substrates, selectively promoting leukemic stem cell death while sparing normal hematopoietic progenitors (139).

**Figure 7:**
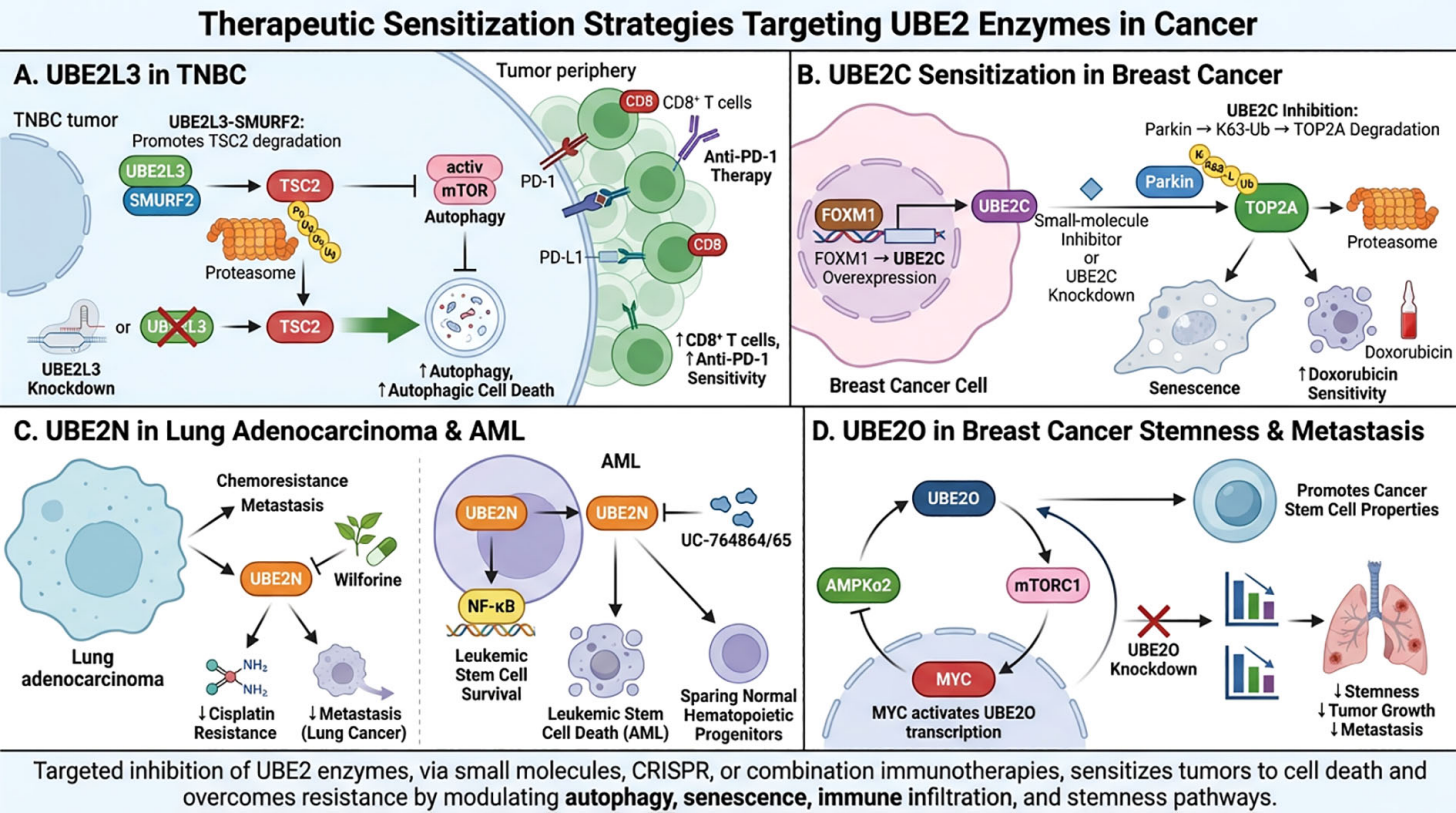
UBE2 enzymes represent versatile therapeutic sensitization targets that enhance tumor vulnerability to chemotherapy, immunotherapy, and combination treatment strategies. Through regulation of autophagy, senescence, immune infiltration, chemoresistance, leukemic stem cell survival, and cancer stemness, targeting UBE2L3, UBE2C, UBE2N, and UBE2O restores treatment responsiveness and supports rational combination approaches across multiple malignancies.

At the intersection of metabolic and stemness-driven resistance, UBE2O sustains breast cancer stem cell properties through a self-reinforcing UBE2O/AMPKα2/mTORC1–MYC feedback loop, where MYC transcriptionally promotes UBE2O expression; UBE2O knockdown suppresses tumor growth and lung metastasis in xenograft models (140). These converging findings highlight UBE2 enzymes as candidate sensitization targets, with proposed strategies ranging from natural compounds and small-molecule inhibitors to combination approaches with immune checkpoint blockade (**Figure 7**). An important and increasingly emphasized dimension of the biomarker literature reviewed here is its convergence with immune-stratification frameworks. UBE2-based prognostic signatures are not merely correlates of proliferation or stage; multiple pan-cancer analyses now associate UBE2C, UBE2S, UBE2T, and UBE2N expression with tumor-mutational burden, immune-cell infiltration patterns, and predicted response to immune checkpoint blockade (28, 88, 90, 141, 142). Read together with the immune-evasion mechanisms reviewed earlier, these preliminary biomarker associations from retrospective analyses support a unified framework that requires prospective confirmation in which UBE2 expression operates simultaneously as a tumor-intrinsic prognostic readout and as an immune-microenvironment correlate, motivating composite signatures that integrate tumor-cell expression with immune-microenvironment features rather than expression alone.

## 8 Prognostic and diagnostic biomarker applications across tumor types

The mechanistic insights into UBE2 enzyme function summarized throughout this review provide a plausible biological foundation for exploring their potential as prognostic and diagnostic biomarkers. However, it is critical to emphasize that the majority of reported associations derive from retrospective analyses of public databases (primarily TCGA and GEO), relatively modest immunohistochemistry (IHC) cohorts, or single-institution series. These findings should therefore be regarded as hypothesis-generating and correlative rather than definitive. Independent validation in large, prospective, multi-center cohorts with standardized assays, rigorous multivariable adjustment for established clinicopathological variables, and functional qualification of the signatures will be required before any UBE2-based biomarker can be considered for clinical implementation (**Table 6**).

**Table 6:** Prognostic and diagnostic biomarker applications of UBE2 family members across tumor types.

UBE2 enzyme	Cancer type	Prognostic direction	Key clinical associations	Immune/TME correlation	Validation approach	Ref.
UBE2C, UBE2T, UBE2S	HCC	Poor OS	All three upregulated across stages 1–4; higher in stages 2–3 vs. 1; associated with TP53 mutation	—	TCGA bioinformatics	(143)
UBE2T	HCC	Poor OS	Upregulated in tissues and cell lines; regulated by miR-212-5p; enhanced proliferation and migration	—	Microarray, IHC, dual-luciferase	(144)
UBE2C	HCC	Poor OS (HR = 1.870)	Higher tumor grade and later stage; correlated with cell cycle proteins (PPI)	Increased immunosuppressive molecules	TCGA, GEO, HPA; independent predictor	(145)
UBE2C	CCA	Poor OS, DSS, LRFS, MFS	Correlated with surgical margin, histological variants, and grade	—	Transcriptome (GSE26566), IHC	(146)
UBE2C	Gastric (intestinal-type)	Poor OS	Associated with CIN; 20q copy number gain in intestinal-type lines; ERK pathway activation (reversible by U0126)	—	Multicenter IHC (n=1,868), microarray	(147)
UBE2N	LUAD	Poor OS	Independent prognostic factor; elevated in advanced stages; promotes immune evasion and drug resistance	Low-UBE2N: NK↑, DC↑, effector T↑, antigen presentation↑	Multi-cohort, CRISPR screening, TMA, IHC	(141)
UBE2H	LUAD	Poor OS	Hypoxia-mediated gene; upregulated in metastatic pleural tumor; correlated with CNV	Inversely associated with miR-101, miR-30a/b, miR-328, miR-497	RNA-seq (matched primary/metastatic), knockdown	(148)
UBE2C	Prostate	Poor prognosis	Knockdown inhibited proliferation, migration, invasion, and xenograft growth; cell cycle arrest and apoptosis induction	—	TCGA, GEO, cBioPortal, COSMIC; xenograft	(149)
UBE2C	UCEC	Poor OS	Most significantly overexpressed UBE2 member; correlated with stage, histological subtype, menopause status, TP53 mutation; DNA hypomethylation and amplification	—	Systematic UBE2 family analysis; *in vitro*	(150)
UBE2J1	HGSOC	Poor OS (upon overexpression)	Plasma cell-related marker; promotes proliferation, invasion, EMT (Slug, Twist1, E-cadherin modulation)	Plasma cells interact with CD8+ T, fibroblasts, endothelial cells	scRNA-seq, LASSO Cox, *in vivo*	(151)
UBE2D3	Esophageal	Favorable OS	Downregulated in tumor tissues; inversely correlated with hTERT; independent prognostic factor (multivariate)	—	IHC (n=150 tumor, 30 normal)	(152)
UBE2S	ESCC	Poor OS (HR = 1.685)	Independent prognostic factor; correlated with ethnicity and vessel invasion; UBE2S+/HIF1a+ = worst prognosis	—	IHC (n=259), TCGA, GEO; multivariate Cox	(153)
UBE2S, HIF-1a, FOXM1	ESCC	Poor OS and PFS	UBE2S: ethnicity, tumor location; HIF-1a/FOXM1: lymph node metastasis; triple co-overexpression = poorest outcome	—	IHC (LIN, HIN, ESCC, controls); Spearman correlation	(154)
UBE2B	ESCA	Poor OS	Upregulated; involved in NF-kB, EMT, inflammatory response, hypoxia pathways; high sensitivity and specificity	CD8+ T↑, Th1↑, macrophages↑; Tem↓, Th17↓	TCGA univariate Cox, knockdown validation	(155)
UBE2C	ESCC	Diagnostic (SMD = 1.98; AUC = 0.93)	Highly expressed at protein and mRNA levels (772 ESCC vs. 1,837 controls); stage-dependent roles; ceRNA: HCP5/miR-139-5p/UBE2C	—	In-house RNA-seq, IHC, TCGA-GTEx, TMA; pooled SMD, sROC	(156)
UBE2T	ESCC	Poor OS	Increased transcript levels and DNA copy numbers; associated with p53, cell cycle, FA pathway	Low-risk group more sensitive to chemotherapy	TCGA, GEO, Oncomine; LASSO Cox; IHC (n=90)	(157)
UBE2T	MM	Poor PFS	Protein progressively increases across R-ISS stages I-II-III; positively correlated with B2-MG and LDH; low-expression: 87.18% vs. high: 71.05% 1-year PFS	—	IHC bone marrow biopsy (n=77 MM, 16 controls)	(163)
UBE2S	Osteosarcoma	Poor OS	Overexpressed; impacts tumorigenesis pathways and treatment sensitivity; knockdown reduces proliferation/migration, promotes apoptosis	Immune landscape correlation	TCGA, GEO; nomogram; transcriptome, IHC; *in vivo*	(164)
UBE2S	SKCM	Poor OS	Highly expressed at mRNA and protein; correlated with proliferation, invasion, metastasis, cell cycle, quiescence at single-cell level	Strong correlation with immune infiltration in TME (P<0.001)	Multi-database (GEPIA2, HPA, CellTracer, TISIDB); single-cell	(175)
UBE2C	Thyroid	Dual (shorter DFS; enhanced invasion upon KD)	High expression predicts shorter DFS; knockdown inhibits proliferation but enhances migration/invasion and sorafenib resistance	Positive correlation with immune infiltrates	TCGA; *in vitro* knockdown; pathway analysis	(165)
UBE2T, ANLN	Breast	Poor OS	Both upregulated; Th1/Th2 balance shifted toward Th2 in Basal and Luminal-B subtypes	Th1↓, Th2↑ in Basal/Luminal-B	GEO; TIMER database; survival analysis	(166)
UBE2S, UBE2C	Breast	Poor OS	Both overexpressed; higher in advanced grade/stage/poor survival; cooperatively downregulate Numb; HR+ BC: lower UBE2S/UBE2C, higher Numb	—	Oncomine, CCLE, HPA, qRT-PCR, WB; overexpression/knockdown	(167)
UBE2C	Breast	Poor OS (independent factor)	Differentially upregulated; correlated with PDCD1, CD274, CTLA4; positive correlation with TMB and MSI	B↓, CD4+ T↓, CD8+ T↓, macrophages↓, myeloid DC↓	GEO, TCGA, qRT-PCR (n=37); TIMER; GSEA (786 immune gene sets)	(168)

### 8.1 Gastrointestinal and hepatobiliary cancers

In HCC, multiple UBE2 family members have been associated with prognosis in retrospective analyses. A comprehensive analysis of UBE2C, UBE2T, and UBE2S revealed that all three are overexpressed across all four disease stages, with significantly higher expression in stages 2–3 compared to stage 1; overexpression of each gene has been correlated with reduced overall survival, and patients harboring TP53 mutations exhibit further elevated UBE2 expression levels, suggesting a possible functional link between p53 inactivation and UBE2 dysregulation (143). UBE2T upregulation in HCC, validated by microarray and immunohistochemistry and regulated by miR-212-5p, has been associated with unfavorable prognosis and enhanced malignant phenotypes (144). UBE2T upregulation in HCC, validated by microarray and immunohistochemistry and regulated by miR-212-5p, has been associated with unfavorable prognosis and enhanced malignant phenotypes (144). UBE2C has similarly been correlated with advanced tumor grade, later stage, and shorter survival (HR = 1.870); notably, UBE2C overexpression is associated with increased immunosuppressive molecule expression (145).

Beyond HCC, UBE2C has shown associations with prognosis across the hepatobiliary spectrum. In cholangiocarcinoma, high UBE2C expression has been associated with adverse surgical margins, advanced histological grade, and reduced overall, disease-specific, local recurrence-free, and metastasis-free survival (146). In gastric cancer, UBE2C has been linked to the intestinal-type subtype associated with chromosomal instability; copy number gains at 20q are observed in intestinal-type but not diffuse-type cell lines, UBE2C overexpression activates ERK signaling reversible by U0126 inhibition, and elevated UBE2C protein has been correlated with poor clinical outcomes in a multicenter cohort of 1,868 cases (147). These findings, while promising, are derived primarily from retrospective cohorts and should be interpreted with appropriate caution.

### 8.2 Thoracic, urological, and gynecological malignancies

Associations between UBE2 family members and prognosis have been reported across thoracic, urological, and gynecological cancers. In LUAD, elevated UBE2N expression has been correlated with advanced disease stage, enhanced immune evasion signatures, and reduced responsiveness to both immunotherapy and targeted therapies in retrospective multi-cohort analyses. Low-UBE2N tumors have been reported to exhibit elevated NK cell, dendritic cell, and effector T-cell infiltration alongside enhanced antigen presentation (141). UBE2H, a hypoxia-responsive gene, has been associated with metastatic pleural disease in LUAD and EMT signaling; its expression correlates with copy number variation and is inversely associated with a panel of protective microRNAs including miR-101, miR-30a/b, miR-328, and miR-497 (148). In urological malignancies, UBE2C expression has been correlated with proliferation, migration, invasion, and xenograft tumor growth in prostate cancer models (149).

Among gynecological cancers, UBE2C has been reported as one of the most significantly overexpressed members of the UBE2 family in uterine corpus endometrial carcinoma (UCEC), correlating with advanced stage, histological subtype, menopausal status, and TP53 mutation (150). In high-grade serous ovarian cancer, single-cell RNA sequencing has identified UBE2J1 as a plasma cell-associated gene that promotes proliferation, invasion, and EMT through modulation of Slug, Twist1, and E-cadherin expression (151). These associations, although suggestive, derive largely from retrospective datasets and require prospective validation.

### 8.3 Esophageal and head-and-neck cancers

Esophageal squamous cell carcinoma (ESCC) and esophageal carcinoma (ESCA) present examples of how multiple UBE2 family members have been investigated as potential complementary biomarkers within a single tumor type. UBE2D3 expression has been reported to be lower in tumor tissues compared to adjacent normal mucosa and inversely correlated with human telomerase reverse transcriptase (hTERT) levels; multivariate analysis identified UBE2D3 as a favorable prognostic factor alongside lymph node status and tumor location (152). In contrast, UBE2S has been associated with adverse prognosis in ESCC (HR = 1.685); co-expression of UBE2S and HIF-1α has been linked to the poorest prognosis (153). The association of UBE2S is further supported by its co-overexpression with HIF-1α and FOXM1 in ESCC, where elevated levels of all three proteins correlate with reduced overall and progression-free survival (154).

UBE2B represents another oncogenic biomarker in ESCA, with high expression correlating with NF-κB, EMT, and inflammatory pathways, as well as altered immune cell composition characterized by increased CD8^+^ T-cells, Th1 cells, and macrophages alongside decreased effector memory T-cells and Th17 cells (155). UBE2C has shown diagnostic associations in ESCC, with a pooled standardized mean difference of 1.98 and an sROC area under the curve of 0.93 across 772 ESCC and 1,837 control samples (156). UBE2T has been associated with p53 signaling, Fanconi anemia, and cell cycle pathways; prognostic gene signatures derived from UBE2T-associated genes have been used to stratify patients into risk groups with differential chemotherapy sensitivity (157). These observations suggest that combinatorial assessment of UBE2 family members may enhance prognostic precision, although all current evidence remains retrospective and requires prospective confirmation.

### 8.4 Hematological malignancies as a distinct translational context for UBE2 biology

Although UBE2 cancer literature is dominated by solid tumors, emerging evidence from leukemias, myelodysplastic syndromes, and plasma cell malignancies suggests that hematological cancers may represent a mechanistically distinct context for UBE2 biology, with potential implications for clinical translation. In acute myeloid leukemia (AML), UBE2N has been investigated as a relevant UBE2 target. Mechanistically, UBE2N catalyzes K63-linked polyubiquitination that supports TRAF6-dependent innate immune signaling and may stabilize leukemia-associated proteomic programs through engagement of the TRIM21 E3 ligase, with downstream effects preferentially involving the immunoproteasome (139, 158, 159). Notably, conditional catalytic-dead C87S knock-in mouse models indicate that UBE2N catalytic activity is required for leukemic maintenance while being relatively dispensable for normal hematopoiesis (159), suggesting a potential therapeutic window that is further supported by the leukemia-selective activity of the UC-764864/UC-764865 inhibitor series in preclinical models (139). In parallel, UBE2C overexpression has been reported to sustain AML proliferation through PI3K/AKT activation and ferroptosis suppression (82), identifying an additional AML-relevant axis that may merit evaluation in combination-based strategies.

Chronic myeloid leukemia (CML) provides complementary insights into UBE2 biology beyond canonical proliferative regulation. Recurrent somatic UBE2A loss-of-function mutations have been detected during progression from chronic phase to blast crisis at approximately 17% frequency, where they impair UBE2A catalytic activity and disrupt myeloid differentiation programs (160). These findings suggest that UBE2A alterations may contribute to clonal evolution rather than act as initiating lesions. In a mechanistically distinct axis, UBE2Q1 is upregulated in BCR-ABL T315I-mutant CML cells, where it stabilizes DDX3 through aberrant ubiquitination. Bortezomib-mediated depletion of UBE2Q1 has been shown to sensitize T315I tumors to anti-PD-1 blockade in experimental models (161). Together, these observations indicate that CML may provide a useful setting in which both loss-of-function and gain-of-function UBE2-related mechanisms can be explored, although their therapeutic relevance requires further validation.

In myelodysplastic syndromes, GATA1-driven UBE2O contributes to proteomic remodeling during terminal erythroid differentiation, raising the possibility that pharmacological modulation of UBE2O could improve ineffective erythropoiesis in erythropoietin- or luspatercept-refractory settings; however, this concept remains largely preclinical (162). In multiple myeloma, UBE2T protein abundance on routine bone marrow biopsy increases with R-ISS stage and has been associated with inferior progression-free survival (163), supporting its potential value as a biopsy-accessible prognostic biomarker complementary to established cytogenetic classifiers. Collectively, these hematological examples suggest that the structural accessibility of leukemic and plasma cells, existing biomarker-driven risk-stratification frameworks, and possible leukemia-selective vulnerabilities may make hematological malignancies a promising, but still incompletely validated, setting for early-phase clinical evaluation of UBE2-targeted strategies.

### 8.5 Dermatologic, bone, thyroid, and breast tumors

The biomarker applications of UBE2 family members appear to extend to mesenchymal, endocrine, and epithelial malignancies, suggesting potential cross-tumor relevance, although the strength of evidence varies across tumor types and study designs. In osteosarcoma, UBE2S overexpression has been associated with poor prognosis and has been linked to tumorigenesis-related pathways, immune landscape features, and treatment sensitivity. A nomogram incorporating UBE2S showed prognostic utility in the analyzed cohort, while UBE2S knockdown reduced proliferation and migration and promoted apoptosis in experimental models, supporting its potential functional relevance; nevertheless, further validation in larger and independent clinical cohorts is warranted (164). Thyroid carcinoma provides a particularly instructive example of the context-dependent roles of UBE2 enzymes. UBE2C knockdown inhibits proliferation and induces apoptosis, consistent with an oncogenic function, yet it has also been reported to enhance migration, invasion, and sorafenib resistance, suggesting that UBE2C may exert dual or stage-dependent effects. Pathway analyses indicate that metabolism-related pathways are enriched in UBE2C-low tumors, whereas cell growth- and immune-related pathways predominate in UBE2C-high tumors; these observations should be interpreted as hypothesis-generating until further mechanistic and clinical validation is available (165).

In breast cancer, several UBE2 members have been proposed to provide complementary prognostic information. UBE2T upregulation, together with ANLN, has been associated with a shift in the Th1/Th2 balance toward Th2 polarization, particularly in basal and luminal-B subtypes, suggesting possible subtype-specific immune prognostic relevance (166). UBE2S and UBE2C have been reported to cooperatively downregulate the cell fate determinant Numb, and a combined UBE2S/UBE2C-high and Numb-low signature was associated with poorer prognosis in ER-positive breast cancer, indicating a potential combinatorial biomarker strategy that requires additional validation (167). UBE2C expression has also been correlated with immune checkpoint genes, including PDCD1, CD274, and CTLA4, as well as tumor mutational burden and microsatellite instability, raising the possibility that it may contribute to immune-related prognostic stratification or prediction of immunotherapy response; however, such applications remain preliminary and should be confirmed in prospective clinical settings (168). Beyond the UBE2 family, the deubiquitinase USP2 functions as a negative regulator of gastric cancer, with low expression associated with adverse prognosis and altered immune infiltration; a USP2-derived genomic model reliably predicts patient outcomes and drug sensitivity (169). Collectively, these findings suggest that UBE2 enzymes, either individually or as part of combinatorial biomarker panels, may represent biologically grounded prognostic candidates across multiple human malignancies, while emphasizing the need for larger cohorts, standardized methodologies, and prospective validation before their routine clinical application.

## 9 Therapeutic targeting strategies and pan-cancer perspectives

The mechanistic and biomarker evidence summarized above provides a biological rationale for considering UBE2 enzymes as candidate pharmacological targets. It must be emphasized at the outset, however, that the field remains at an early preclinical stage: the relatively flat and conserved active site of E2 enzymes has historically complicated selective drug design, and although recent advances in structure-based screening, allosteric inhibitor discovery, and natural-product pharmacology have generated several preclinical tool compounds, none has yet entered registered clinical evaluation as a UBE2-targeted agent in oncology. The candidates discussed in the following subsections should therefore be regarded as proof-of-concept demonstrations of pharmacological tractability rather than as clinically validated therapies, and the broader caveats outlined in our Critical Appraisal section regarding selectivity profiling, pharmacodynamic biomarkers, and prospective clinical validation apply directly to the literature reviewed below.

### 9.1 Current state of clinical development

An honest appraisal of the current pharmacological landscape requires explicit acknowledgement that, despite the expanding preclinical literature, the clinical development of UBE2-directed agents remains nascent. To our knowledge, no selective inhibitor of any UBE2 family member has yet advanced through registered clinical trials as a UBE2-targeted oncology indication. The compounds discussed throughout this review fall into three categories that should be carefully distinguished. First, dedicated preclinical UBE2 modulators, including HA-9104 (UBE2F) (170), piperine (UBE2T) (171), cephalomannine (UBE2S) (109), pentagalloylglucose (UBE2T) (89), NSC697923 (UBE2N) (85), and UC-764864/65 (UBE2N) (139), remain at the *in vitro* and animal-model stage, with selectivity against the broader UBE2 superfamily and pharmacodynamic engagement biomarkers incompletely characterized. Second, repurposed agents such as leflunomide (115) and 2-deoxy-D-glucose (120) are clinically established for non-oncology or metabolic indications and have shown UBE2-relevant activity in preclinical cancer models, but their clinical use as UBE2-pathway therapeutics is hypothesis-generating rather than evidence-based. Third, indirect modulators, most notably CDK4/6 inhibitors, which incidentally suppress UBE2C/S/T expression in ER-positive breast cancer (79), are clinically approved for unrelated mechanisms, and the contribution of UBE2 downregulation to their clinical efficacy remains unproven and likely partial.

Recognizing this developmental stage has direct implications for how the therapeutic claims throughout this review should be interpreted. References to “therapeutic exploitation,” “translatable strategies,” or “combination approaches” indicate biological plausibility supported by preclinical data, not clinically demonstrated benefit. Translating any of these candidates to patient care will require, at minimum, validated target engagement assays, selectivity profiling against the full ubiquitin–conjugation machinery, formal toxicology in models that recapitulate the physiological roles of UBE2 enzymes in normal tissues, and prospective clinical trials with biomarker-stratified patient selection. We revisit these requirements in greater detail in the Critical Appraisal section.

### 9.2 Small-molecule inhibitors and natural compounds

Translating UBE2 biology into therapeutic intervention has advanced through both structure-based drug design and natural product screening. HA-9104, discovered via virtual screening and chemical optimization, represents the first small molecule selectively targeting the neddylation E2 enzyme UBE2F. HA-9104 binds UBE2F, reduces its protein levels, and consequently inactivates cullin-RING ligase 5 (CRL5), leading to accumulation of the pro-apoptotic CRL5 substrate NOXA and induction of apoptosis. Additionally, HA-9104 induces DNA damage and G2/M arrest through DNA adduct formation via its 7-azaindole moiety. Biologically, HA-9104 demonstrates antitumor activity as a single agent and confers significant radiosensitization in lung cancer xenograft models, establishing proof-of-concept for selective UBE2 E2 enzyme targeting (170).

Natural compounds offer complementary pharmacological strategies. Piperine, an alkaloid derived from black pepper, allosterically modulates UBE2T within the Fanconi anemia pathway, blocking FANCL-mediated ubiquitin transfer to the FANCI/FANCD2 complex without disrupting E2 charging or FANCL–UBE2T binding affinity; differential scanning fluorimetry confirms direct UBE2T engagement. Combined piperine and cisplatin treatment significantly reduces bladder cancer xenograft growth by selectively impairing interstrand crosslink repair while leaving intrastrand adduct processing intact (171).

At the pathway intersection level, genome-wide CRISPR resistance screens in pancreatic ductal adenocarcinoma uncovered a mutual and targetable codependence between PI3Kα/δ and SUMOylation signaling (166). Simultaneous pharmacological inhibition triggers synthetic lethality, reduces tumor growth, and promotes immune cell infiltration in syngeneic orthotopic immune-competent models. These findings provide a compelling rationale for combining PI3K and SUMO-pathway inhibitors in clinical translation (172).

### 9.3 Metabolic and synthetic lethality-based interventions

Metabolic vulnerabilities created by UBE2 enzyme dysregulation offer promising therapeutic opportunities grounded in synthetic lethality principles. In pancreatic cancer, UBE2T catalyzes RING1-mediated p53 ubiquitination, relieving transcriptional repression of ribonucleotide reductase subunits M1/M2 and driving unrestrained pyrimidine biosynthesis that alleviates replication stress and confers gemcitabine resistance. Conditional Ube2t knockout in spontaneous KPC mice markedly extends survival following gemcitabine treatment, and UBE2T expression positively correlates with gemcitabine resistance in clinical specimens. High-throughput compound library screening using patient-derived organoids identified pentagalloylglucose (PGG) as a potent UBE2T inhibitor and gemcitabine sensitizer; the PGG–gemcitabine combination diminishes tumor growth in patient-derived xenograft models and prolongs survival in spontaneous pancreatic cancer mice, supporting further translational evaluation as an approach to overcome gemcitabine resistance (89).

The neddylation E2 enzyme UBE2F presents a parallel targetable vulnerability specifically in KRAS-mutant pancreatic cancer. In the KrasG12D mouse model, Ube2f deletion suppresses cerulein-induced pancreatitis, acinar-to-ductal metaplasia, and pancreatic intraepithelial neoplasia progression by inactivating MAPK–c-Myc signaling through stabilization of the tumor suppressor DIRAS2, a substrate of the CRL5^ASB11^ E3 ligase complex. DIRAS2 deletion largely rescues the phenotypes induced by Ube2f loss, supporting an epistatic relationship within this oncogene–tumor suppressor cascade and strengthening the rationale for targeting the UBE2F–CRL5ASB11 axis. UBE2F and DIRAS2 expression levels may additionally serve as prognostic biomarkers for patient stratification in KRAS-driven pancreatic adenocarcinoma (173).

### 9.4 Pan-cancer landscape and cross-tumor therapeutic opportunities

Pan-cancer analyses provide the integrative framework necessary to translate the tumor-specific observations described throughout this review into broadly applicable therapeutic strategies. UBE2T is highly expressed and functions as a prognostic risk factor across the majority of human cancers, with single-cell sequencing confirming a strong positive correlation with cell cycle activity (r > 0.60). In lung adenocarcinoma, UBE2T-related cell cycle gene signatures classify patients into four molecular subtypes with significantly different survival, immune landscapes, and predicted immunotherapy responsiveness; immunologically “hot” subtypes exhibit higher immunophenoscores and greater likelihood of responding to immune checkpoint blockade (142).

UBE2S similarly demonstrates pan-cancer oncogenic properties, with elevated expression unfavorably associated with clinical stage and prognosis across nearly all tumor types analyzed. UBE2S positively correlates with myeloid-derived suppressor cells and Th2 subsets while conferring immune evasion through co-expressed immunoinhibitors and T-cell exhaustion markers. Higher UBE2S expression is also associated with elevated stemness, tumor mutational burden, microsatellite instability, mismatch repair deficiency, and chemotherapeutic resistance, supporting its potential utility as a multifaceted immune-oncogenic biomarker (28).

Circulating EPAS1 mRNA in plasma from colorectal cancer patients associates with disease relapse and reduced survival in advanced stages, illustrating the potential of liquid biopsy approaches incorporating ubiquitin-pathway transcripts (174). The UBE2O/AMPKα2/mTORC1–MYC positive feedback loop drives proliferation, EMT, and cancer stemness in breast cancer, with pharmacological disruption of this self-reinforcing circuit suppressing tumor growth and lung metastasis in xenograft models (140). Collectively, these pan-cancer observations suggest that UBE2 enzymes function not only as individual candidate targets but also as interconnected nodes within broader ubiquitin regulatory networks that may be therapeutically exploitable across multiple tumor contexts.

## 10 Critical appraisal: methodological limitations and contradictory findings

The literature reviewed here, while expansive, carries several methodological and conceptual limitations that warrant explicit acknowledgement before broader inferences are drawn. We therefore highlight five caveats that, in our view, define the current ceiling of confidence in the field.

First, contradictory functional assignments among UBE2 family members remain incompletely resolved. The most striking example is UBE2S, which acts as an oncogene in lung adenocarcinoma (103) and hepatocellular carcinoma (106) yet exerts tumor-suppressive activity in colorectal cancer through TRAF6-mediated suppression of both PI3K/AKT and MAPK/ERK signaling (107). UBE2C similarly oscillates between predominantly oncogenic behavior in pan-cancer analyses (88) and tumor-suppressive ferroptosis-related effects in selected gastric cancer contexts (117). Although these divergent outputs are typically attributed to tissue-specific substrate availability, differential E3 partner expression, and microenvironmental cues, direct head-to-head substrate proteomic comparisons across tumor lineages are largely lacking. The functional duality emphasized as a defining theme of this review is therefore *inferred* from non-overlapping studies rather than directly tested in side-by-side experimental systems, a limitation that should temper categorical statements about any single UBE2 enzyme being unambiguously oncogenic or tumor-suppressive. A parallel limitation applies to biomarker studies: while numerous reports describe prognostic associations for specific UBE2 members, these are predominantly based on retrospective TCGA/GEO mining or modest IHC series without adequate multivariable adjustment or independent prospective validation. Consequently, claims of clinical utility remain preliminary.

To make these caveats concrete rather than rhetorical, **Table 7** summarizes representative cases of opposing functional assignments within the UBE2 family and offers candidate reconciliations grounded in tissue context, substrate availability, and chain-type specificity. We emphasize that these reconciliations remain working hypotheses rather than experimentally adjudicated conclusions, and we identify the experimental designs that would be required to test each.

**Table 7:** Representative contradictions in the UBE2 cancer literature and candidate mechanistic reconciliations.

UBE2 enzyme	Oncogenic context	Tumor-suppressive context	Proposed reconciliation	Required experimental adjudication
UBE2S	LUAD: IκBα degradation, NF-κB activation (103); HCC: VHL/HIF-1α and JAK2/STAT3 axis (106, 109)	CRC: TRAF6 ubiquitination, dual PI3K/AKT and MAPK/ERK suppression (107)	Substrate availability and dominant E3 partner differ across lineages; TRAF6 abundance in colonic epithelium may redirect UBE2S output toward kinase-pathway restraint, whereas IκBα and VHL are the dominant accessible substrates in lung and liver contexts.	Lineage-matched isogenic UBE2S knockout panels combined with quantitative substrate proteomics.
UBE2C	Pan-cancer: oncogenic across 33 tumor types, correlated with TMB and stage (88)	Gastric: ACSL4 ubiquitination suppresses ferroptosis, but loss-of-function paradoxically enhances tumor cell death (117)	Apparent contradiction may reflect a phenotype-versus-survival distinction: UBE2C supports proliferation broadly, but in ferroptosis-prone gastric contexts the same enzyme creates a vulnerability rather than an advantage when manipulated.	Conditional UBE2C deletion in genetically engineered gastric models with parallel ferroptosis-pathway profiling.
UBE2D3	PDAC: TAP2 ubiquitination, antigen-presentation blockade, immune evasion (22)	Glioma: SHP-2 activation, STAT3-driven proliferation and glycolysis (105) (oncogenic)	Both are oncogenic but through entirely distinct mechanisms (immune-extrinsic vs. immune-intrinsic), illustrating that “single enzyme/single mechanism” framing is itself a methodological artifact.	Cross-tissue substrate trapping with proximity labeling under matched physiological conditions.
UBE2T	LUAD: cisplatin resistance via autophagy (116); HCC: pyrimidine biosynthesis (115)	No major tumor-suppressive context yet reported	Asymmetric reporting may itself reflect publication bias rather than genuine biological universality.	Systematic loss-of-function screens in low-expressing tissue contexts.

Second, the strength of mechanistic evidence varies considerably across the cited literature. A substantial proportion of studies rely on cell line-based knockdown or overexpression experiments, with comparatively fewer employing patient-derived organoids, conditional knockout models, or pharmacological tool compounds with documented target engagement. Bioinformatic and TCGA-derived correlations, while useful for hypothesis generation, are frequently elevated to confirmatory status despite their well-recognized limitations regarding causality. Several reported E2-substrate interactions also rely on overexpression-based co-immunoprecipitation that may not reflect endogenous, stoichiometric associations under physiological conditions.

Third, *clinical translation remains nascent*. Although prognostic associations between UBE2 expression and patient outcomes are widely reported, multivariable analyses adjusting for established clinicopathological covariates are inconsistently performed, and prospective validation of UBE2-based prognostic signatures in independent cohorts is rare. Pharmacological strategies, including cephalomannine for UBE2S (109) and leflunomide for UBE2T-driven pyrimidine biosynthesis (115), remain largely preclinical, with incomplete selectivity profiling against the broader UBE2 superfamily and limited pharmacodynamic biomarkers of target engagement.

Fourth, publication and reporting biases likely favor positive functional and oncogenic findings. The relative paucity of reports describing UBE2 enzymes with neutral or context-restricted roles, particularly in pan-cancer analyses where most family members appear functionally consequential, may partially reflect selective reporting and statistical thresholds rather than true biological universality.

Fifth, *cross-tumor generalizability of UBE2 mechanisms is more limited than the aggregate literature suggests*. The majority of mechanistic studies have been conducted in a relatively narrow set of tumor lineages, predominantly hepatocellular carcinoma, lung adenocarcinoma, gastric and colorectal cancers, breast cancer, and pancreatic ductal adenocarcinoma, while many other malignancies, including most hematologic cancers, sarcomas, central nervous system tumors, and rare gastrointestinal subtypes, remain comparatively underrepresented. Within the studied lineages, the same UBE2 enzyme may engage entirely distinct substrates, E3 partners, and downstream pathways, as illustrated by UBE2D3’s tumor-intrinsic immune-evasion role in PDAC (22) versus its STAT3-mediated proliferative role in glioma (105). Statements presented elsewhere in this review under the umbrella of “across diverse malignancies” or “in cancer” should therefore be interpreted as referring to the specific tumor types in which the underlying primary studies were performed, rather than as universal claims about UBE2 biology in cancer broadly. Where we have used such umbrella phrasing, it reflects the convergent direction of available evidence rather than mechanistic equivalence across tissue contexts.

Recognizing these caveats does not diminish the central messages of this review; rather, it clarifies the boundaries within which current evidence should be interpreted. The convergence of mechanistically distinct studies on shared signaling and immunometabolic nodes, and on the tumor–immune interface, suggests that the broad themes outlined here are unlikely to be artifacts of any single methodological approach. Nonetheless, decisive progress will require systematic E2–substrate proteomic mapping in disease-relevant tissue contexts, conditional genetic models that recapitulate native expression dynamics, and prospective clinical validation of biomarker signatures before UBE2-targeted strategies can be confidently advanced to routine clinical practice.

## 11 Prioritization framework: ranking UBE2 family members by functional, clinical, and therapeutic evidence

The breadth of associations reported across the UBE2 family does not readily translate into actionable guidance regarding which members merit prioritized investigation. We therefore propose a transparent three-axis framework integrating: (i) *functional evidence strength*, mechanistic depth, substrate diversity, independent replication, and *in vivo* confirmation (76, 88–92, 100, 103, 112, 113); (ii) *clinical and biomarker evidence*, tumor-type breadth, cohort consistency, and multivariable validation (28, 88, 90, 141, 142, 146, 147); and (iii) *therapeutic tractability*, dedicated chemical probes, structural amenability, and demonstrated pharmacodynamic engagement (85, 89, 109, 139, 170, 171). Application to twelve UBE2 members most extensively represented in cancer literature yields the stratification in **Table 8**, assigning each enzyme to one of three priority tiers.

**Table 8:** Three-axis prioritization of representative UBE2 family members based on functional, clinical, and therapeutic evidence.

UBE2 enzyme	Functional evidence (cancer-relevant)	Clinical/biomarker evidence	Therapeutic tractability	Priority tier	Most informative starting context
UBE2C	Extensive: APC/C cofactor, mitotic driver, PI3K/AKT–PD-L1 axis, Notch and KRAS regulation (76, 92–94)	Strong pan-cancer prognostic signal across 33 tumor types (88); HCC, cholangiocarcinoma, gastric cancer cohorts (146, 147)	Moderate: indirect suppression by approved CDK4/6 inhibitors (79); no selective inhibitor yet	Tier 1	Pan-cancer biomarker; combination with CDK4/6 blockade in ER-positive breast cancer
UBE2T	Extensive: Wnt/β-catenin, Akt/β-catenin, autophagy, pyrimidine metabolism, FA pathway (91, 95, 115, 116)	Pan-cancer prognostic risk factor (142); immunotherapy-response stratification in LUAD (142)	Highest among UBE2 family: piperine (171), pentagalloylglucose (89), leflunomide repurposing (115)	Tier 1	HCC and PDAC; gemcitabine and cisplatin combinations
UBE2S	Extensive: APC/C function, NF-κB, VHL/HIF-1α, JAK2/STAT3, ferroptosis suppression (103, 106, 109, 119)	Pan-cancer adverse prognostic and immune-evasion signal (28, 90)	Emerging: cephalomannine in preclinical HCC models (109)	Tier 1	HCC immunometabolic axis; ovarian platinum-resistance
UBE2N	Substantial: MEK/FRA1/SOX10 in melanoma, oncogenic innate immune signaling in AML, glycolysis in prostate cancer (85, 114, 139)	Lung adenocarcinoma prognostic signature (141); AML biomarker context (139)	High: NSC697923 (85), UC-764864/65 (139), wilforine (138)	Tier 1	Melanoma and AML; LUAD chemoresistance
UBE2D3	Moderate–strong: TAP2/MHC-I axis in PDAC, SHP-2/STAT3 in glioma (22, 105)	Emerging: PDAC immune-evasion biomarker (22)	Limited: no dedicated inhibitor; conceptually attractive for combination with TCR-T therapy	Tier 2	PDAC immune sensitization
UBE2I (UBC9)	Moderate–strong: SUMOylation-dependent macrophage polarization, p27 nuclear export (121, 122, 135)	Ovarian and thyroid cancer microenvironment data (121, 122)	Moderate: SUMO pathway inhibitors under broader development	Tier 2	Ovarian cancer combination with anti–PD-L1
UBE2O	Moderate: AMPKα2/mTOR/HIF-1α axis, breast cancer stem cell circuit (87, 140)	Breast and prostate cancer mechanistic data (87, 140)	Limited: no dedicated chemical probe yet	Tier 2	Breast cancer stemness biology
UBE2F	Moderate: neddylation E2, NOXA-dependent apoptosis, platinum resistance (129, 170)	Lung adenocarcinoma context (129, 170)	Notable: HA-9104 as a selective preclinical inhibitor (170)	Tier 2	Lung cancer radiosensitization
UBE2L3	Limited–moderate: TSC2/mTOR/autophagy axis, anti-PD-1 sensitization (136)	Single TNBC mechanistic study (136)	Limited: no dedicated chemical probe	Tier 3	TNBC immunotherapy combination (hypothesis-generating)
UBE2L6	Limited: ABCB6-mediated cisplatin resistance (125)	Single resistance-cohort study (125)	Limited: no dedicated chemical probe	Tier 3	Cisplatin-resistant tumor models
UBE2D2	Limited: CST1/GPx4 axis in gastric ferroptosis (118)	Gastric cancer mechanistic data (118)	Limited: no dedicated chemical probe	Tier 3	Ferroptosis-pathway research
UBE2H	Limited: single-cell PDAC immune-suppression study (123)	Exploratory single-cell data (123)	Limited: no dedicated chemical probe	Tier 3	PDAC immunology research

Several cross-axis patterns emerge. First, mechanistic centrality and biomarker breadth correlate strongly within Tier 1: UBE2C, UBE2T, UBE2S, and UBE2N each combine pan-cancer prognostic signal with multiple validated mechanistic substrates (28, 88, 139, 141, 142). Second, mechanistic depth does not predict tractability, UBE2C, despite occupying the highest evidence density, lacks a selective inhibitor and is targeted only indirectly through approved CDK4/6 inhibitors (76), whereas UBE2T benefits from three independently developed preclinical strategies (89, 115, 171). Third, several Tier 2 enzymes anchor mechanistically distinctive but quantitatively narrow evidence: UBE2D3 defines a unique PDAC immune-evasion axis (22) but lacks comparable depth elsewhere, while UBE2I integrates SUMOylation-dependent macrophage and T-cell regulation (121, 122) without UBE2I-selective probes. Fourth, Tier 3 enzymes (118, 123, 125, 136) each anchor interesting findings supported by single-laboratory or single-cohort evidence requiring replication.

For *researchers*, the framework facilitates target prioritization: Tier 1 enzymes warrant translational mechanistic and combination preclinical work, Tier 2 enzymes, particularly UBE2D3 and UBE2I, offer disproportionate scientific return from dedicated chemical probe development, and Tier 3 enzymes invite discovery-oriented investigation. For *clinicians and translational investigators*, the convergence of Tier 1 status with disease-specific evidence concentrations, UBE2C and UBE2S in HCC (92, 94, 106, 109), UBE2T in HCC and PDAC (89, 91, 95, 115), and UBE2N in melanoma and AML (85, 139), identifies contexts where biomarker-guided patient stratification is most defensible.

We caution that all priorities remain anchored in preclinical and observational data, and that the broader translational caveats discussed in caveat 3 of the Critical Appraisal section apply uniformly across all tiers. The contradiction-reconciliation analysis in Table X further qualifies the Tier 1 assignments for UBE2S and UBE2C, both exhibiting context-dependent functional duality (104, 114) that should refine rather than negate their prioritization.

## 12 Limitations in clinical translation of UBE2-targeted therapies

Despite the compelling preclinical rationale outlined in the preceding sections, the translation of UBE2-targeted strategies into clinical practice faces a series of substantive obstacles that merit explicit acknowledgment, particularly as this field begins to interface with drug-development pipelines. The same caution applies to biomarker applications: although UBE2 expression patterns show promising correlations with outcome and immune features in retrospective datasets, robust prospective qualification is still required.

### 12.1 Druggability and selectivity constraints

Members of the UBE2 family share a highly conserved ~150-residue ubiquitin-conjugating (UBC) fold and a catalytic cysteine embedded in a structurally similar active-site environment across more than thirty human paralogs. The relative absence of deep, well-defined ligand-binding pockets and the predominantly flat protein–protein interaction surfaces have historically placed this family among the so-called “undruggable” targets. The most advanced chemical probes against individual UBE2 enzymes, including the natural product–derived UBE2N inhibitor reported in lung adenocarcinoma (138), the UBE2S-targeting strategy in hepatocellular carcinoma (109), the virtual-screening–derived UC-764864/UC-764865 series targeting UBE2N (139), and HA-9104 targeting the UBE2F–CRL5 axis (170), remain at the lead-optimization or preclinical proof-of-concept stage, with limited pharmacokinetic characterization and no entry into registered Phase I oncology trials. The broader cautionary precedent of upstream ubiquitin/NEDD8 pathway inhibitors, several of which advanced into randomized clinical evaluation but failed to deliver definitive survival benefit, underscores the substantial gap that persists between elegant target validation and reproducible clinical efficacy. Emerging modalities including covalent inhibitors, proteolysis-targeting chimeras (PROTACs), and molecular glues offer pragmatic alternative entry points but remain dependent on E2–E3 cognate partnerships that are incompletely mapped.

### 12.2 Functional duality complicates patient stratification

As consolidated in **Tables 1** and **7**, multiple UBE2 family members, most notably UBE2S, UBE2D3, UBE2J1, UBE2M, and UBE2C, exhibit lineage-dependent functional duality, operating as oncogenes in some tumor contexts and as tumor suppressors in others. Indiscriminate pharmacological inhibition of a dual-role enzyme in an indication where it operates as a tumor suppressor could plausibly accelerate, rather than restrain, malignant progression, an underappreciated risk for first-in-human trials enrolling histology-agnostic basket cohorts. Bulk-tissue mRNA or immunohistochemical abundance, the most widely used surrogates for “dependency” in the current literature, correlate imperfectly with enzymatic activity, E3 partner availability, and substrate accessibility within a given tumor. Robust translation will therefore require activity-based or substrate-specific companion diagnostics, analogous to those used for kinase and PARP inhibitors.

### 12.3 The evidence base remains predominantly preclinical and retrospective

Most functional data summarized in this review derive from RNA-interference– or CRISPR-based perturbation in cell lines and subcutaneous xenografts, with comparatively few studies in orthotopic models, patient-derived xenografts, or genetically engineered mouse models. Prognostic associations rest disproportionately on retrospective interrogation of TCGA and GEO datasets without independent multi-cohort validation or harmonized scoring criteria. The absence of any completed Phase II or III trial powered on UBE2 expression or activity precludes formal estimation of clinical effect sizes, and meta-analytic statements about UBE2 prognostic signatures should presently be regarded as hypothesis-generating rather than confirmatory.

### 12.4 On-target/off-tumor toxicity and immune homeostasis

Several UBE2 enzymes prioritized as oncology targets, including UBE2N, UBE2D3, and UBE2J1, also fulfill indispensable housekeeping functions in DNA-damage response, ER-associated degradation, and innate immune signaling in non-malignant tissues. Systemic catalytic inhibition therefore carries the risk of dose-limiting hematological toxicity, impaired antiviral immunity, and disruption of proteostasis in metabolically active organs. Because several UBE2 enzymes additionally modulate antigen presentation, T-cell activation, and tumor-associated macrophage polarization (22, 122, 123, 136), broad pharmacological inhibition could paradoxically antagonize, rather than synergize with, concomitant immune-checkpoint blockade, an outcome opposite to that suggested by tumor-restricted genetic ablation studies. Tumor-selective delivery, conditional or inducible PROTACs, and rigorous longitudinal monitoring of peripheral immune compartments during early-phase trials will be essential.

### 12.5 Resistance and combinatorial complexity

Because UBE2 enzymes operate within an interconnected E2–E3 network with substantial functional redundancy, monotherapy with selective UBE2 inhibitors is unlikely to achieve durable responses; alternative E2 paralogs may compensate, or upstream E3 ligases may redirect activity through bypass pathways. Most preclinical efficacy reported in the therapeutic studies summarized in **Table 5** has been observed in combination with chemotherapy, radiotherapy, or immune-checkpoint blockade rather than as single-agent activity (129, 130, 136–138, 170). Rational combination design will require systematic mapping of UBE2–E3 dependencies, prospective identification of compensatory enzymes that emerge under sustained UBE2 inhibition, and pharmacological strategies that pre-empt rather than chase emerging resistance.

Taken together, these limitations do not diminish the therapeutic promise of UBE2 enzymes but rather delineate the translational work that remains. The path forward will require concerted progress across three convergent fronts: medicinal chemistry capable of producing selective covalent inhibitors, PROTACs, and molecular glues; translational biomarker development yielding activity- and substrate-based companion diagnostics with prospective qualification; and biomarker-stratified, indication-specific, combination-aware clinical trial design.

## 13 Conclusions and future perspectives

In summary, this review consolidates an expanding but still fragmented body of literature into three integrative concepts that we view as the principal conceptual advance of the present synthesis over earlier UBE2-focused reviews. First, beyond their canonical role as ubiquitin carriers, several UBE2 enzymes contribute, together with their E3 partners and substrate context, to substrate engagement and ubiquitin chain-type selection in ways that are biologically meaningful in cancer; importantly, the magnitude and direction of this contribution vary considerably across family members and remain incompletely defined for several UBE2 enzymes, a limitation that should temper broad generalizations about UBE2 ‘activeness’. Second, the most distinctive theme emerging from recent literature is *functional duality*: the same UBE2 member can drive or restrain tumor progression depending on tissue lineage, substrate repertoire, E2–E3 partnership, and microenvironmental context, which directly challenges single-function annotations frequently adopted in earlier reviews. Third, multiple structurally and mechanistically distinct UBE2 members converge on a relatively small set of shared signaling and immunometabolic nodes—including PI3K/AKT, MAPK/ERK, NF-κB, HIF-1α, antigen-presentation machinery, and checkpoint-ligand regulation, suggesting that UBE2-targeted strategies may exert disproportionate biological effects through pathway-level convergence rather than through any single enzyme’s intrinsic ‘centrality’.

From a translational perspective, the most promising implications of UBE2 biology lie in its intersection with immunopharmacology. Increasing evidence indicates that UBE2 dysregulation contributes to defective antigen presentation, checkpoint ligand expression, macrophage polarization, regulatory immune-cell induction, and resistance to systemic therapy, thereby providing a rationale for combination strategies with chemotherapy, targeted agents, ferroptosis-inducing approaches, and immune checkpoint blockade. At the same time, clinical translation will require greater mechanistic resolution and caution. Specifically, we emphasize that the field is currently at a preclinical and proof-of-concept stage; no UBE2-directed agent has yet entered registered clinical evaluation as a UBE2-targeted indication, and the existing therapeutic literature, although biologically compelling, should be read as hypothesis-generating rather than as clinically validated. Decisive translational progress will require systematic mapping of E2–E3–substrate networks in disease-relevant tissue contexts, prospective validation of UBE2-based prognostic and predictive biomarkers in independent cohorts, development and clinical qualification of pharmacodynamic markers of UBE2 target engagement, selectivity profiling across the full UBE2 superfamily, and rigorous toxicology that accounts for the physiological roles of these enzymes in normal tissues. With these caveats acknowledged, UBE2 enzymes should be viewed as mechanistically grounded but clinically unproven candidate nodes at the interface of cancer biology and tumor immunity, whose realistic near-term contribution to patient care lies primarily in biomarker-guided stratification, while their potential as direct therapeutic targets awaits the dedicated clinical evaluation that the current evidence base does not yet provide.
